# MTFNet: A Lightweight Multi-Scale Time–Frequency Neural Network for Multi-Class Event Recognition in DAS-Based Perimeter Security

**DOI:** 10.3390/s26206398

**Published:** 2026-10-10

**Authors:** Qiankun Zhang, Yangyang Sun, Zhilong Chen, Linjian Ma, Xiaoyu Zhao, Kai Cheng, Haoning Li

**Affiliations:** 1 College of Field Engineering, Army Engineering University of PLA, Nanjing 210007, China; zhang_qk37117@126.com (Q.Z.); haoningli03@126.com (H.L.); 2 College of National Defense Engineering, Army Engineering University of PLA, Nanjing 210007, China; chen-zl@vip.163.com (Z.C.); patton.4400@163.com (L.M.); 3 College of Engineering and Applied Sciences, Nanjing University, Nanjing 210023, China; 15152443300@163.com; 4 College of Command and Control Engineering, Army Engineering University of PLA, Nanjing 210007, China; chengkai911@aeu.edu.cn

**Keywords:** DAS, event recognition, MTFNet, perimeter security, noise robustness

## Abstract

Distributed Acoustic Sensing (DAS) has emerged as a pivotal technology for perimeter security. However, existing DAS event recognition methods have inherent limitations. Single-domain modeling has limited feature representation, while conventional multi-domain fusion methods require converting one-dimensional signals into two-dimensional images. This conversion adds computational overhead and may discard part of the original phase and amplitude information. In addition, the robustness of lightweight recognizers under low signal-to-noise ratio (SNR) conditions remains insufficiently examined. This paper proposes a lightweight multi-scale time–frequency network (MTFNet) for direct processing of one-dimensional DAS signals. The model adopts a parallel time–frequency dual-branch architecture. The time-domain branch utilizes a lightweight 1D Inception-ResNet variant to capture short-term impulse features and long-range temporal dependencies via multi-scale parallel convolutions and residual connections. The frequency-domain branch performs multi-scale segmented spectral analysis to extract complementary discriminative information at diverse time and frequency resolutions, followed by a lightweight attention-free fusion module for effective time–frequency feature fusion. Experimental results on a self-built dataset covering Wind, Facility Noise, Hammering, Sawing, and Walking events show that MTFNet achieves a recognition accuracy of 99.55% with 0.12 M parameters, outperforming SVM, 1D-CNN, TCN, MS 1D-CNN, CNN-BiLSTM, and attention-based multimodal fusion methods. It maintains an accuracy of 94.06% at an SNR of 0 dB, demonstrating its robustness against additive white Gaussian noise (AWGN) and practical engineering application potential.

## 1. Introduction

Growing demands for perimeter safety monitoring have arisen for critical infrastructures. To be specific, the infrastructures encompass oil–gas pipelines, railway corridors, transformer substations, as well as national border zones. In general, conventional perimeter protection solutions cover video surveillance, infrared beam sensors and electronic fences [1,2]. Nevertheless, the above-mentioned approaches exhibit drawbacks in long-range continuous detection, harsh environment adaptability and electromagnetic interference resistance. Consequently, they can hardly meet the requirements of modern intelligent security systems for all-weather, long-distance and highly reliable monitoring.

Distributed Acoustic Sensing (DAS), typically implemented via phase-sensitive optical time-domain reflectometry (Φ-OTDR), stands out with prominent merits (e.g., corrosion resistance, anti-electromagnetic interference capability, superior detection sensitivity, fast response and long laying distance) [3]. In recent years, this technique has been widely applied in multiple research fields, ranging from seismic prospecting [4], road traffic condition monitoring [5,6] and pipeline leakage inspection [7,8,9] to perimeter security warning [3,10]. In addition to achieving precise spatial localization of intrusion-related activities, the DAS system can automatically classify event categories with the assistance of feature extraction and pattern recognition algorithms. It thus provides essential technical support for intelligent early warning and active perimeter defense.

Early studies on DAS-based perimeter intrusion recognition mostly adopted classic machine learning algorithms, namely the support vector machine (SVM) [11], k-nearest neighbors (KNN) [12] and random forest (RF) [13]. The classification rules of these algorithms rely heavily on manually designed feature engineering and professional domain expertise. Although these models maintain decent interpretability, they involve cumbersome data preprocessing workflows.

The extracted features are mostly shallow statistical features, which can hardly fully characterize the deep discriminative information contained in DAS signals under complex scenarios. Accordingly, the generalization ability of the model is significantly limited in such scenarios [14].

To address the above-mentioned limitations, deep learning methods have been widely applied in the field of DAS intrusion recognition in recent years by virtue of their end-to-end automatic feature learning capability. Wu et al. [8] performed noise reduction on raw time-domain signals, adopted a one-dimensional convolutional neural network (1D-CNN) for feature extraction, and implemented classification of five typical oil pipeline signal categories using an SVM. This workflow achieved an average accuracy of 96%. Nevertheless, single-size convolution kernels fail to fully capture multi-level signal features under complex working conditions. To address this limitation, Wu et al. [15] proposed a multi-scale one-dimensional convolutional neural network (MS 1D-CNN), in which parallel convolution kernels of different sizes are deployed to extract multi-scale temporal features. Another study by Wu’s research group [16] presented a hybrid multi-scale convolutional neural network–hidden Markov model (mCNN-HMM) architecture. This framework effectively explores the correlations between multi-scale feature maps and enhances the overall performance of feature extraction. Xu et al. [17] constructed a hybrid network integrating the Gramian angular difference field (GADF), cross-attention fusion module (CAFM), and multi-scale convolutional neural network (MSCNN). The cross-attention unit enhances information interaction among multi-scale feature branches.

In terms of multi-domain fusion, Wang et al. [18] combined a long short-term memory (LSTM) network and a CNN. Taking time-domain curves and their short-time Fourier transform (STFT) results as inputs, the model extracts temporal features as well as contour and energy features of signals, achieving accuracies of 97.8% and 94.6% on the training set and test set for four types of signals, respectively. Shi et al. [19] processed raw spatial–temporal signals through band-pass filtering and grayscale transformation to generate spatial–temporal matrix data. Their method achieved a recognition accuracy of 96.67% for five types of disturbance events. Zhang et al. [20] constructed a dual-channel parallel network integrating 1D-CNN and 2D-CNN to extract time-domain and frequency-domain features from original signals and STFT spectrograms, respectively. Furthermore, a multi-layer Transformer encoder structure with a multi-head self-attention mechanism was adopted for multimodal feature fusion, which achieved a recognition accuracy of 98.54% on the urban pipeline dataset.

Hu et al. [21] proposed a time–frequency fusion convolutional neural network (TFF-CNN) architecture that adopts two-dimensional time–frequency representations, including GADF and fast Fourier transform (FFT) spectra. A residual network (ResNet) backbone was used to extract deep-level features, and a decision-level fusion module mimicked human-like judgment logic. This system achieved an average classification accuracy of 99.30% for five signal categories. Bai et al. [22] presented a dual-branch spatial–temporal synergy network (DB-STSNet). Three core modules, namely the Feature Spatial–Temporal Attention Fusion (F-STAF), Feature Temporal Channel Attention Fusion (F-TCAF), and Dual-branch Multi-scale Deep-level Cross Fusion (DM-DCF) modules, enable adaptive fusion between one-dimensional temporal features and two-dimensional spatial–temporal data. A noise-aware cross-entropy (NA-CE) loss function was specially designed to improve model robustness under strong ambient noise interference.

According to the above research results, two representative technical paths have been proposed to boost the classification accuracy of DAS event detection: multi-scale modeling and multi-domain feature fusion. At present, the existing methods have several defects. On the one hand, single time-domain networks can only capture short-term impulse responses and long-range temporal dependencies to a limited extent, and the frequency-domain pattern and energy distribution characteristics are not fully utilized. Reliable classification performance cannot be guaranteed for multiple intrusion events with very similar time-domain waveforms based solely on temporal information. At the same time, most prevalent multi-domain fusion methods map the one-dimensional signal to a two-dimensional form, such as STFT spectrograms and GADF images. Feature extraction and subsequent fusion are generally carried out by 2D-CNN, Transformer or attention-based modules. The above operations add model parameters and computational overhead, and may result in some loss of the original phase and amplitude information of the sensing signal. Transformer and multi-head attention modules are computationally expensive, so they are not suitable for lightweight design and real-time hardware deployment of DAS detection models. Finally, although noise robustness has begun to draw attention in recent DAS recognition studies [22], it is seldom assessed alongside model compactness. Lightweight recognizers are typically validated only on clean or field data, whereas noise-oriented studies rarely quantify the trade-off between robustness and model complexity.

Given the above deficiencies, this paper proposes a lightweight MTFNet for DAS-based perimeter security event recognition. The proposed framework directly processes the one-dimensional phase-difference signal demodulated from the DAS system and employs a dual-branch structure of time-domain and frequency-domain branches for multi-scale discriminative feature extraction. The design can improve classification accuracy by avoiding the computation-heavy generation of 2D time–frequency maps. The primary contributions of this paper are as follows:

(1) A time–frequency dual-branch architecture is designed that takes one-dimensional signals as the direct input. Unlike existing DAS recognition methods that explicitly construct two-dimensional time–frequency spectrograms for 2D neural networks, the proposed architecture extracts features directly from the 1D signal. This approach avoids the explicit spectrogram construction step and reduces the complexity of the subsequent network, thereby facilitating lightweight deployment.
(2) Two supporting multi-scale feature-extraction modules are proposed. In the time-domain branch, a modified 1D Inception-ResNet structure is adopted, in which parallel multi-scale convolutions with kernel sizes of 3, 7, and 15 combined with residual connections jointly capture short-term impulse features and long-range temporal dependencies. In the frequency-domain branch, three-scale sliding-window spectrum analysis with window lengths of 128, 256, and 512 is performed to extract complementary discriminative information at diverse temporal and frequency resolutions.
(3) A lightweight attention-free time–frequency fusion module is designed. High-cost fusion schemes such as Transformer and multi-head attention are avoided, and feature interaction is realized merely through linear mapping, batch normalization, and nonlinear activation. The proposed model achieves a recognition accuracy of 99.55% with only 0.12 M parameters.

## 2. DAS System and Dataset

### 2.1. System Configuration and Working Principle

**Figure 1:**
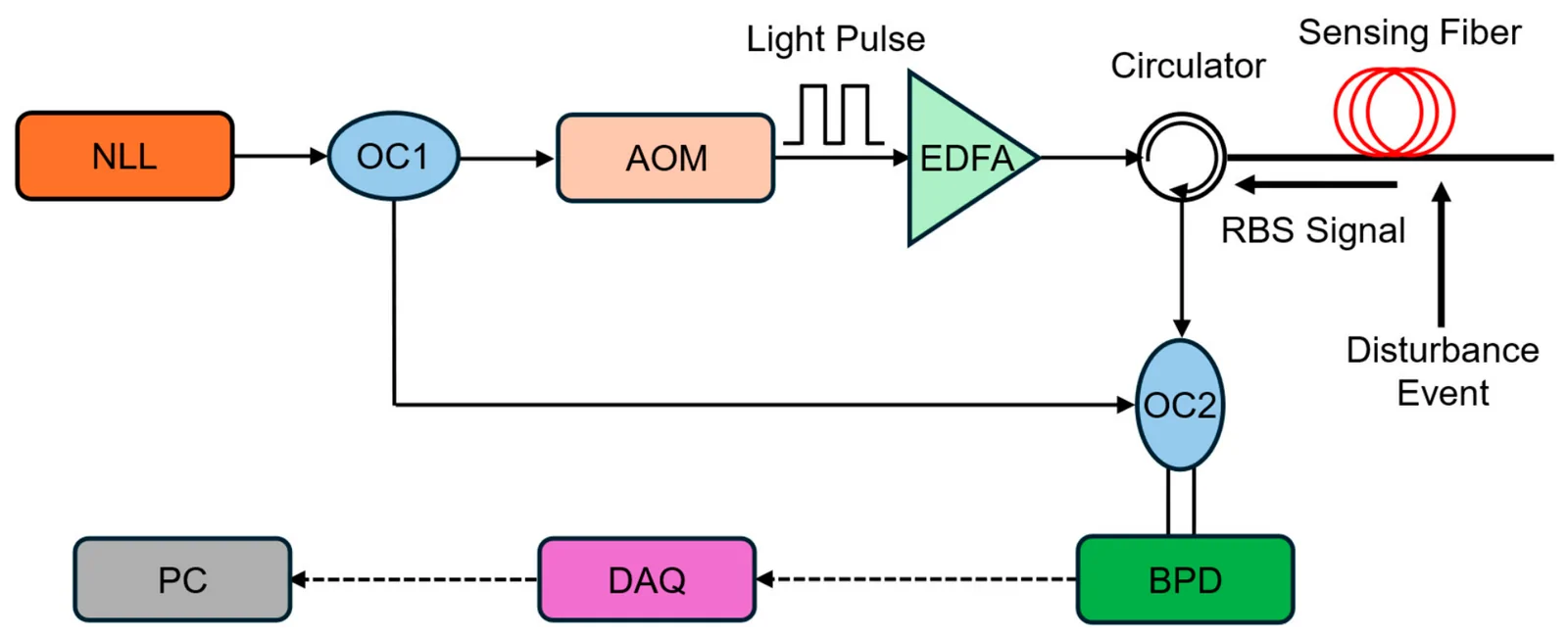
Schematic diagram of the DAS system. Solid arrows represent optical paths, and dashed arrows represent electrical connections.

Figure 1 depicts the DAS system built on the Φ-OTDR scheme for the experiments in this paper. Its hardware components include a narrow-linewidth laser (NLL), optical coupler (OC), acousto-optic modulator (AOM), erbium-doped fiber amplifier (EDFA), optical circulator, sensing optical fiber, balanced photodetector (BPD), data acquisition card (DAQ), and host processing unit. The operational workflow is described as follows. Continuous coherent light emitted by the NLL is split into two beams by the OC. One beam is converted into pulsed light via the AOM, amplified by the EDFA, and guided into the sensing fiber through the optical circulator to generate Rayleigh backscattering (RBS) within the fiber. The other light beam acts as the local reference. After beam coupling inside the OC, the two optical signals are transmitted to the BPD for optoelectronic conversion. The acquired electrical waveform is subsequently transferred to the host computer through the high-speed DAQ for subsequent signal processing. When external disturbances are applied to the optical fiber, variations in the refractive index, length, and core diameter at the corresponding fiber location occur, resulting in phase changes in the RBS signal. Two fiber segments before and after the disturbed zone are selected as reference regions. The phase difference between the two reference segments varies linearly with the disturbance applied to the fiber segment between the two reference regions [23]. For the round-trip backscattered light, this relation can be written as:

(1) $$∆\varphi =\frac{4\pi n_{f}}{\lambda }\Delta L$$

where *λ* denotes the wavelength of the incident laser in vacuum, *n_f_* is the refractive index of the fiber core, and *ΔL* represents the change in length of the fiber segment between the two reference positions, induced by acoustically driven axial strain. Specifically, a strain *ε* = *ΔL*∕*L_g_* (where *L_g_* is the distance between the two reference segments) gives rise to an optical-path change of *n_f_*⋅*∆L* for the one-way propagation. Because the backscattered light traverses the segment twice, the round-trip optical-path change becomes 2*n_f_*⋅*∆L*, which corresponds to the phase difference *∆φ* in Equation (1). Equation (1) thus relates the phase variation linearly to the axial strain of the fiber segment. Therefore, by demodulating the phase variations in the backscattered signal, the *Δφ* can be obtained, enabling the localization and recognition of disturbance events.

The main parameters of the DAS system are as follows: a central wavelength of 1550.12 nm, a laser linewidth of 3 kHz, a pulse width of 50 ns, a pulse repetition frequency of 1 kHz, and a spatial sampling interval of 2.5 m.

### 2.2. Dataset Preparation

**Figure 2:**
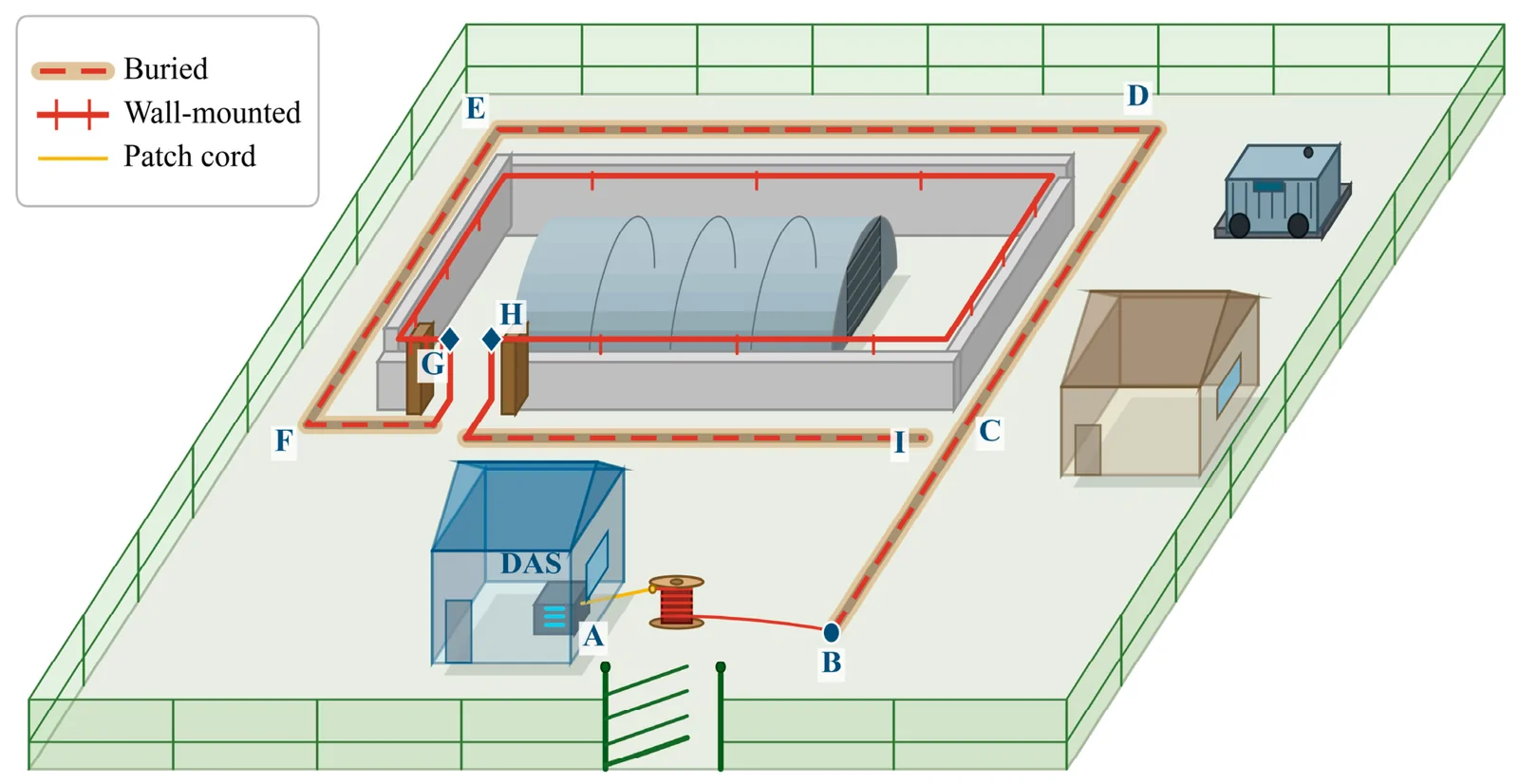
Schematic diagram of the sensing optical cable deployment at the experimental site. The dashed and solid red lines denote the buried and wall-mounted cable sections, respectively, and the yellow line denotes the patch cord; letters A–I mark the key locations along the cable route described in the text.

The field experiment was conducted at a guarded site in an open field in Inner Mongolia, China. The site is enclosed by a perimeter wall, with a gate on the south side. The DAS interrogator was installed in an equipment room at the center of the site, while the cable reel was placed on the outdoor ground east of the equipment room. The sensing optical cable, which incorporates two single-mode fibers, had a total length of approximately 700 m. Roughly 220 m remained on the reel, and the remaining 480 m served as the sensing section. As illustrated in Figure 2, one end of the cable was connected to the output port of the interrogator through a jumper (point A). The other end was led out from the reel to point B, where the cable changed from an exposed layout to underground-buried installation. The buried section was laid in an excavated trench outside the perimeter wall. It passed successively through points C, D, E, and F to the gate area (point G), where the cable was switched to wall-mounted installation. The cable then ran clockwise along the inner surface of the perimeter wall for approximately one full circle to point H near the gate. It passed through the wall and resumed buried installation outside the wall, and extended to the terminal point I. For the buried section, the covering soil depth was about 0.3 m, and the backfilled soil was moderately compacted. The wall-mounted section was fixed onto the inner surface of the perimeter wall.

**Figure 3:**
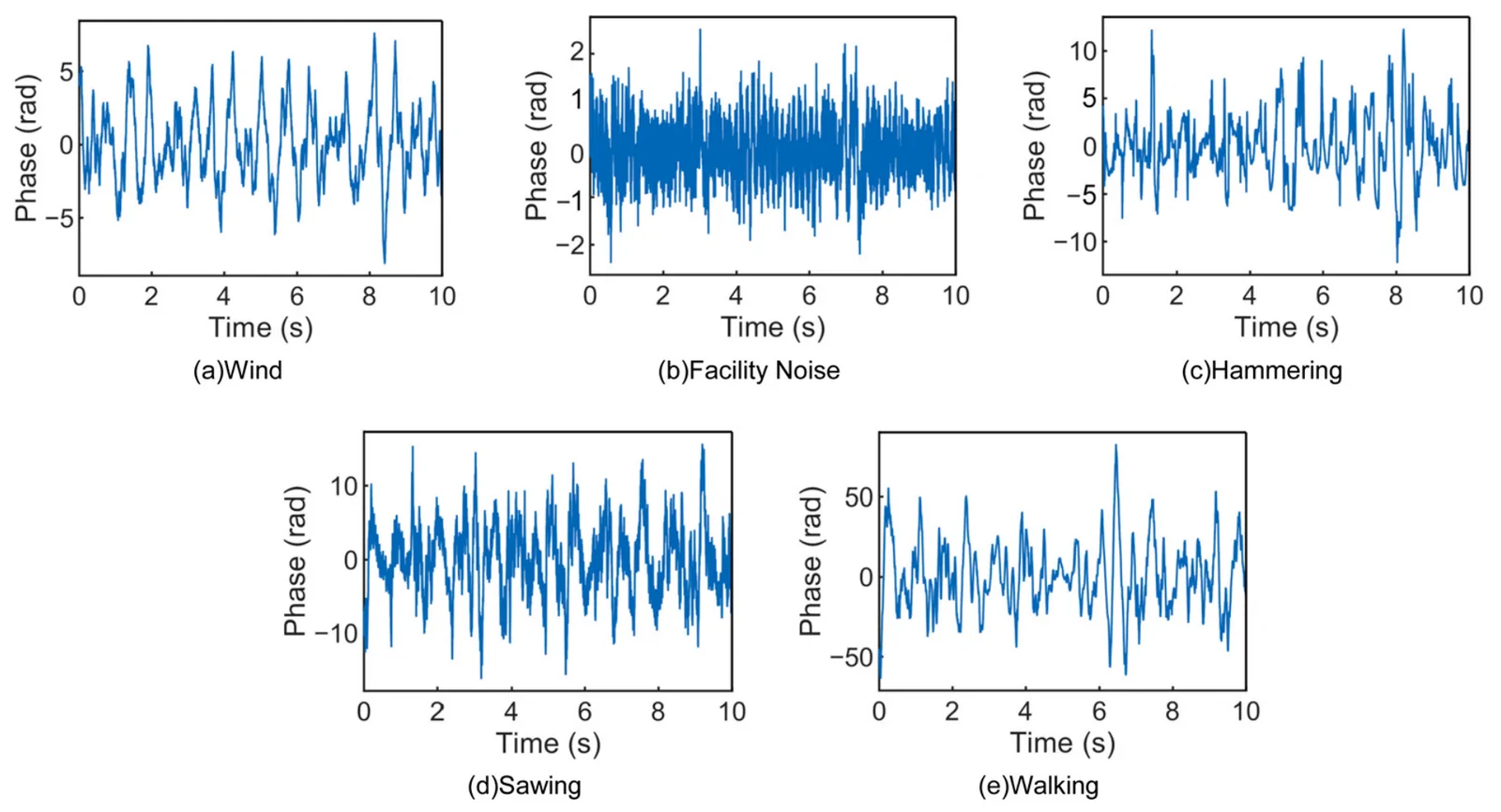
Temporal waveforms of the five event categories. (**a**) Wind; (**b**) Facility Noise; (**c**) Hammering; (**d**) Sawing; (**e**) Walking.

Five typical signal types were collected during the experiments, namely wind-induced vibration, facility noise, hammering, sawing, and walking events. Among them, hammering, sawing, and walking were regarded as typical intrusion events, whereas wind-induced vibration and facility noise were considered the primary environmental disturbances. The five categories were produced as follows. (1) Wind: natural wind-induced vibrations of the perimeter wall were collected at different times of the day under varying wind strengths along all wall-mounted sections. (2) Facility Noise: steady-state vibrations generated by a continuous operation of a mobile power station installed on the northeastern side of the perimeter wall were recorded in the wall-mounted sections. (3) Hammering: impact destruction on the wall or facilities was simulated using three to four different demolition tools on the wall-mounted sections. (4) Sawing: continuous cutting destruction was performed with saws or functionally similar tools (three to four types) along all wall-mounted sections. (5) Walking: four operators with different heights and weights (165 cm/60 kg, 170 cm/68 kg, 176 cm/75 kg, and 185 cm/87 kg) walked along all buried sections at speeds of approximately 1, 1.5, and 2 m/s, under both loaded and unloaded conditions. Representative temporal waveforms of the five event categories are shown in Figure 3.

Data acquisition was carried out over approximately one month. For each event category, experiments were repeated across multiple sessions covering different periods of the day (early morning, morning, noon, afternoon, and evening). These sessions were performed by different operators at different positions along the sensing fiber. To prevent information leakage among the training, validation, and test sets, the dataset was partitioned at the acquisition-session level rather than at the frame level. Specifically, all frames segmented from the same session were assigned to exactly one subset, ensuring that frames from the same physical event realization never appear in more than one subset. The demodulated phase-difference time series of a single spatial channel served as the basic recognition unit. Within each session, the signal segments containing the disturbance events were first excerpted from the continuous recordings. These segments were then divided into non-overlapping frames to generate fixed-length samples, without any sliding-window overlap. A frame length of 2 s was adopted because it covers the complete temporal evolution of all five event types, including the intervals between consecutive hammering strikes and the gait cycle of walking. Thus, each sample is a single-channel one-dimensional sequence containing 2000 time-step points. The frame-level segmentation produced 5492 wind samples, 5635 facility noise samples, 5125 hammering samples, 4676 sawing samples, and 5377 walking samples, yielding an overall training/validation/test ratio of approximately 7:1:2 under the session-independent partition. Sample statistics for each category are summarized in Table 1. Acquisition statistics of each category, including the numbers of sessions, positions, and operators, are summarized in Table 2. No manual denoising was applied to the samples, so that the evaluation reflects the model’s performance under realistic noise conditions.

It should be noted that the two fiber installations were arranged to enrich the coupling conditions of the dataset rather than to be evaluated separately, and the samples from both installations were pooled together; since the network input is a single-channel sequence without any positional identifier, the model cannot directly exploit deployment or location labels. The facility noise was recorded in the wall-mounted sections, whose coupling path runs from the power station through the ground and the wall; its recognition relies mainly on the stable narrowband spectral pattern of steady-state operation, rather than on location-specific coupling features. In addition, treating background disturbances (wind, facility noise) and intrusion events (hammering, sawing, walking) as separate classes is a simplification of real scenarios, where background and intrusion vibrations may be superimposed.

Due to the large dynamic range of vibration signal amplitudes collected by the DAS system, significant differences exist in the energy levels among different events. Therefore, a linear normalization method is adopted to map the signal amplitudes into the range of [−1, 1], thereby mitigating the dimensional discrepancies caused by different acquisition conditions. The corresponding computational formula is expressed as:

(2) $$x_{\mathrm{norm}}=\frac{2\left(x-x_{\min}\right)}{x_{\max}-x_{\min}}-1$$

where *x* denotes the demodulated phase-difference sequence of one sample, *x*_min_ and *x*_max_ denote the minimum and maximum values of the corresponding signal segment, and *x*_norm_ indicates the normalized signal. The normalization is applied before the samples are fed into the network.

## 3. Network Architecture Design

### 3.1. Overall Framework of the Proposed Network

**Figure 4:**
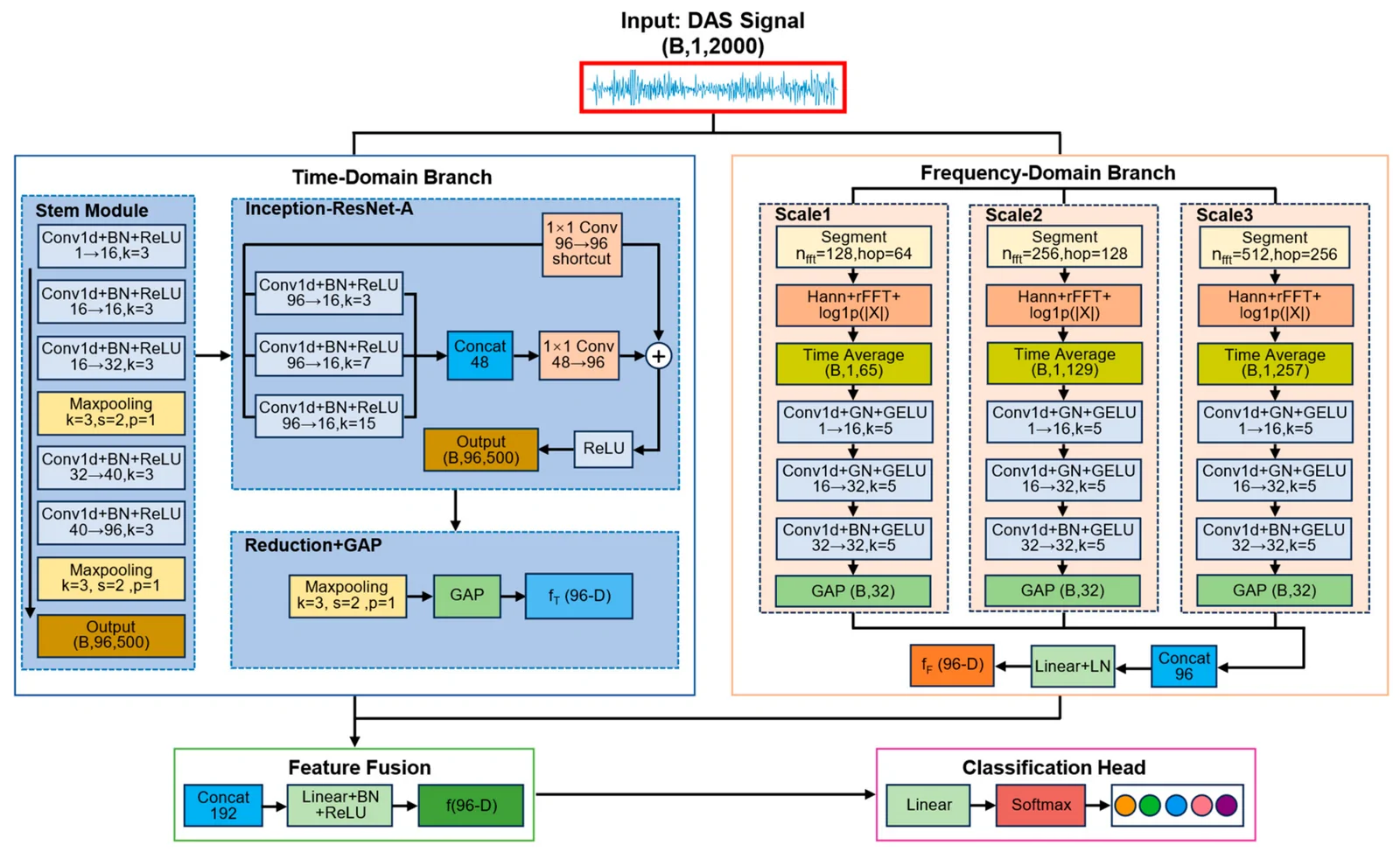
Overall architecture of MTFNet. The four shaded regions denote the functional modules, i.e., the time-domain branch, frequency-domain branch, feature fusion, and classification head; blocks of the same colour represent the same type of operation across modules (e.g., light blue for 1-D convolution, yellow for max pooling, green for global average pooling (GAP) and linear projection, and bright blue for concatenation), and the five coloured circles at the output denote the five event categories.

Constrained by the complex time–frequency characteristics of DAS perimeter security events and the limited classification performance of single-domain modeling, a lightweight multi-scale time–frequency network, termed MTFNet, is proposed in this study. Its complete structure is illustrated in Figure 4. The network accepts the normalized one-dimensional phase-difference sequence, where parallel time-domain and frequency-domain branches extract mutually complementary feature information. A lightweight fusion block implements cross-feature interaction, and the final classification head outputs the predicted intrusion label. The input-sample tensor is defined as *x* ∈ R^B×1×T^, where B represents the batch size and T denotes the time-step length of each individual sample. In the present experiments, T is set to 2000, and the total number of target categories C is equal to 5. The complete forward-propagation workflow of MTFNet is formulated as:

*f_t_* = *F_T_*(*x*) (3)

*f_f_* = *F_F_*(*x*) (4)

*f*_fusion_ = *F_M_*([*f_t_*, *f_f_*]) (5)

*z* = *F_C_*(*f*_fusion_) (6)

Here, *F_T_*(⋅) and *F*_F_(⋅) denote the feature-extraction mappings for the time-domain and frequency-domain branches, respectively; *F*_M_(⋅) represents the time–frequency feature fusion mapping; and *F*_C_(⋅) refers to the classification mapping. *f_t_* and *f_f_* denote time-domain and frequency-domain features, *f*_fusion_ indicates the fused joint feature representation, and z denotes the category scores (logits) output by the classification head. By modeling time-domain and frequency-domain information, MTFNet can strengthen its representation capability for complex vibration patterns and maintain a lightweight model architecture.

### 3.2. Time-Domain Feature-Extraction Branch

The time-domain branch is constructed to capture local temporal variations and multi-scale sequential dependencies embedded in DAS signals. Inspired by Inception-ResNet-V2, a well-known image classification backbone integrating parallel multi-scale convolution and residual shortcut connections [24,25], this study tailors a lightweight one-dimensional variant suitable for one-dimensional waveform processing. Standard Inception-ResNet frameworks are primarily designed for two-dimensional image tasks, and stacking the full network structure would introduce excessive redundant parameters. Conventional 1D-CNNs adopt convolution kernels of a single size, which cannot simultaneously capture sharp impulse shocks and long-period vibration trends. In the modified structure proposed in this study, only the Stem block, one Inception-ResNet A block, and the Reduction A block are retained, while all Inception B, Inception C, and deep-layer Reduction B modules are removed. Parallel 1D convolution kernels with sizes of 3, 7, and 15 are deployed to adapt the network to one-dimensional DAS vibration data. Compared with mainstream temporal sequence networks, the proposed branch possesses substantially fewer trainable parameters. It maintains competitive feature representation performance while reducing overall model complexity. In addition, the multi-scale receptive fields can effectively cover event patterns with different time scales.

1. Stem Block

The Stem block is composed of five cascaded 1D convolutional layers and two max pooling layers. The computational pipeline covers three consecutive convolutional operations, one max pooling operation, another two convolutional operations, as well as a final max pooling operation. Each convolutional layer adopts a kernel size of 3. The layer is coupled with batch normalization and the Rectified Linear Unit (ReLU) activation function. For the max pooling layers, the kernel size and the stride are respectively set to 3 and 2. After data flows through the Stem block, the single input channel tends to expand to 16, 16, 32, 40, and 96. The time sequence length is downsampled from 2000 to 500. This module completes shallow temporal feature extraction and compression along the time dimension.

2. Inception-ResNet A Block

To efficiently extract multi-scale temporal features from one-dimensional waveforms, a customized 1D Inception-ResNet A variant is developed for DAS signals. Three parallel branches are configured with convolution kernels of *k* = 3, *k* = 7, and *k* = 15, each generating feature maps with 16 output channels. The branch equipped with the *k* = 3 kernel provides a small receptive field, which captures transient shocks and sharp local signal mutations. The *k* = 7 kernel branch characterizes vibration patterns with moderate temporal-scale properties, while the *k* = 15 kernel branch with a broad receptive field captures long-term temporal dependencies.

Feature outputs from the three branches are concatenated along the channel axis to form a 48-channel feature tensor. A subsequent 1 × 1 convolution projects the concatenated features into 96 channels. Meanwhile, the input feature undergoes channel dimension matching via an additional 1 × 1 convolution. The aligned shortcut feature is element-wise added to the projected feature, and the ReLU activation function produces the final block output. This structure enhances multi-scale representation capability, while the residual shortcut connection preserves the original temporal information, thereby improving feature expressiveness and maintaining stable network training.

3. Reduction and Global Average Pooling (GAP) Blocks

The Reduction block performs max pooling with a kernel size of *k* = 3 and a stride of 2, further downsampling the sequence length from 500 to 250. Subsequently, GAP compresses the feature map with a shape of (96, 250) into a 96-dimensional vector. The vector serves as the time-domain branch’s final output. We define the mapping function of the time-domain branch as:

*f_t_* = GAP(*Φ_R_*(*Φ_A_*(*Φ_S_*(*x*)))) (7)

where Φ*_S_*(⋅), Φ_A_(⋅), and Φ_R_(⋅) denote the forward mapping operations of the Stem block, Inception-ResNet A block, and Reduction A block, respectively. *f_t_* ∈ ℝ*^B^*^×96^ represents the final 96-dimensional time-domain feature vector.

### 3.3. Frequency-Domain Feature-Extraction Branch

Most existing frequency-domain analysis methods for DAS rely on FFT with a fixed single window length. This choice cannot reconcile the high temporal resolution required for transient shocks with the high frequency resolution required for steady-state vibrations. The frequency-domain branch proposed in this work directly implements three-scale sliding-window FFT spectral extraction on one-dimensional signals. Since spectra are temporally averaged within each scale, this branch delivers a multi-resolution spectral representation instead of a full time–frequency representation. It prioritizes the steady-state spectral structure of events, while the temporal evolution is captured by the time-domain branch. The two branches are thus complementary in their modeling roles.

Three analysis window sizes are configured: W*_k_* ∈ {128, 256, 512}. The corresponding sliding strides are set to 64, 128, and 256, which yields a 50% overlap ratio between adjacent analysis windows. At a 1 kHz sampling rate, the three window lengths of 128, 256, and 512 points correspond to analysis durations of 0.128 s, 0.256 s, and 0.512 s, and yield frequency resolutions of approximately 7.8 Hz, 3.9 Hz, and 2.0 Hz, respectively. Short-length windows capture broadband transient shock components, long-length windows resolve fine-grained frequency structures of periodic steady-state vibration, and medium-sized windows achieve a smooth transition between the two cases.

For the *k*-th analysis scale, *x_k_*_,*τ*_(*n*) represents the *τ*-th frame segmented from the input sequence using the window length W*_k_*, and *w*(*n*) denotes the matched Hann window function. The segmented spectrum formulation is expressed as:

*S_k_*(*τ*, *f*) = log(1 + |rFFT(*w*(*n*) ⋅ *x_k_*_,_*_τ_*(*n*))|) (8)

where rFFT(⋅) denotes the real-valued fast Fourier transform; log(1 + |∙|) compresses the dynamic range of spectral amplitude and helps the network learn the relative energy distribution across distinct frequency bands. Afterwards, spectral outputs of all time-dependent frames under the same analysis scale are averaged along the temporal dimension to generate the scale-aware spectral descriptor:

(9) $$\bar{S_{k}}\left(f\right)=\frac{1}{M_{k}}\sum_{\tau =1}^{M_{k}}S_{k}\left(\tau ,f\right)$$

where *M_k_* denotes the total number of time-domain frames under the *k*-th analysis scale. This operation enables the frequency-domain branch to focus on the steady-state spectral structure of vibration events, which complements the dynamic temporal patterns captured by the time-domain branch.

Three operations in this branch deserve clarification as follows. First, only the magnitude of the real fast Fourier transform (rFFT) output is retained, and the phase spectrum is discarded. This choice is justified because the phase of a short window is highly sensitive to window alignment and noise. In contrast, the discriminative information of steady and periodic vibrations is mainly carried by the magnitude distribution. Second, the log(1 + |∙|) compression reduces the dynamic range of spectral magnitudes. This prevents a few dominant spectral lines from overwhelming the weaker yet discriminative components during training. Third, temporal averaging suppresses instantaneous fluctuations and noise, though it discards the temporal order of the spectra within each scale. This trade-off is deliberate. The temporal order discarded within each scale is not lost at the network level, since it is retained and modeled by the parallel time-domain branch. In other words, the frequency-domain branch is dedicated to stable spectral structures, while the temporal dynamics of events are fully preserved in the time domain. Therefore, the two branches are complementary rather than redundant.

A dedicated one-dimensional convolutional sub-branch is constructed to extract features from the spectral descriptor of each individual scale. Each sub-branch consists of three cascaded 1D convolution layers with a stride of 2 and a kernel size of 5, whose output channel dimensions are sequentially set to 16, 32, and 32. In the frequency-domain branch, each scale-specific sub-branch processes its own spectral descriptor. The effective feature diversity within a mini-batch is lower than that of the input waveform. Batch normalization (BatchNorm) relies on mini-batch statistics, which may become unstable or couple samples within a batch under such conditions. Group normalization (GroupNorm) is therefore adopted in the first two layers, as it computes statistics within each sample independently of the batch composition. The final convolutional layer adopts BatchNorm to align the feature distributions across the three scales before concatenation and fusion. This stabilizes the input distribution of the subsequent fusion block. The Gaussian error linear unit (GELU) is utilized as the unified activation function. Adaptive average pooling generates a 32-dimensional frequency-domain feature vector for each scale:

(10) $$g_{k}=H_{k}\left(\bar{S_{k}}\right)$$

where *H_k_*(⋅) denotes the mapping function of the one-dimensional convolutional sub-branch for the *k*-th scale, and *g_k_* ∈ ℝ*^B^*^×32^ represents the frequency-domain feature extracted at this scale. Features from the three scales are concatenated along the channel dimension to obtain a preliminary 96-dimensional multi-scale frequency-domain feature:

*g* = [*g*_1_, *g*_2_, *g*_3_] ∈ R^B×96^ (11)

The concatenated feature is transformed and normalized via linear projection and layer normalization (LayerNorm), with the aim of producing the final frequency-domain representation:

*f_f_* = LN(*W_f_g* + *b_f_*) (12)

Here, *W_f_* and *b_f_* represent the weight matrix and bias vector of the frequency-domain projection layer, and LN(⋅) denotes the layer-normalization operation. The above procedure represents the complete expansion of the frequency-domain branch mapping *F*_F_(⋅) in Equation (4).

Benefiting from the above-mentioned structure, the frequency-domain branch integrates transient frequency shifts from short-time windows and steady-state spectral patterns from long-time windows within a single network. Consequently, sufficient discriminative spectral information is prepared for the subsequent time–frequency feature fusion.

### 3.4. Time–Frequency Feature Fusion and Classification

In recent years, mainstream time–frequency fusion methods rely on multi-head self-attention and Transformer fusion modules. These components introduce a considerable number of parameters and inference latency, making them less suitable for resource-constrained deployment. To address this issue, this paper designs a minimalist linear fusion unit. It enables time–frequency feature interaction with only a slight increase in parameters. While ensuring feature complementarity, the proposed unit reduces model inference time and is more suitable for the real-time alarm requirements of DAS devices. Specifically, the acquired time-domain and frequency-domain features are concatenated along the feature dimension to construct a 192-dimensional joint time–frequency feature:

*f*_cat_ = [*f_t_*, *f_f_*] ∈ ℝ^B×192^ (13)

To retain time–frequency complementarity, suppress redundant information, and enhance feature interaction, the concatenated feature is fed into a lightweight fusion module. After linear projection, batch normalization, and nonlinear activation, the feature is compressed into a 96-dimensional fused representation:

*f*_fusion_ = ReLU(BN(*W_m_f*_cat_ + *b_m_*)) (14)

where *W_m_* and *b_m_* denote the weight matrix and bias vector of the fusion layer, and BN(⋅) represents the batch normalization operation. The fusion module reduces the feature dimensionality and avoids over-parameterization of the direct classification head, while further enhancing the interaction and representation capability between time-domain and frequency-domain features through nonlinear mapping. The classification head consists of a Dropout layer and a fully connected layer. The former is employed to alleviate overfitting, whereas the latter maps the fused features into the category space.

*z* = *W_c_* ⋅ Dropout(*f*_fusion_) + *b_c_* (15)

where *W_c_* and *b_c_* are the weight matrix and bias vector of the classification layer. The predicted probability that sample i belongs to category c is obtained via the Softmax function:

(16) $${\hat{y}}_{i,c}=\frac{\exp\left(z_{i,c}\right)}{\sum_{c^{\prime }=1}^{C}\exp\left(z_{i,c^{\prime }}\right)}$$

The cross-entropy loss function is adopted during the training phase.

(17) $$L=-\frac{1}{N}\sum_{i=1}^{N}\sum_{c=1}^{C}y_{i,c}\log{\hat{y}}_{i,c}$$

where N denotes the number of samples within a single mini-batch, *C* is the number of categories, and *y_i_*_,*c*_ ∈ {0, 1} is the ground-truth label in one-hot form, and ${\hat{y}}_{i,c}$ is the predicted probability that sample *i* belongs to category *c*, given by the Softmax function in Equation (16).

In summary, MTFNet extracts complementary temporal sequence and spectral features through the time-domain and frequency-domain branches, respectively. Efficient feature interaction is implemented via the lightweight fusion module prior to final event classification. The proposed architecture operates directly on one-dimensional signals, which avoids the explicit construction of two-dimensional time–frequency maps and the associated computational overhead. Despite its compact size, the network exploits multi-scale discriminative cues from both the temporal and spectral domains.

## 4. Experimental Results and Analysis

### 4.1. Experimental Environment and Evaluation Metrics

All experiments are carried out on a workstation fitted with an AMD Ryzen 9 9950X3D central processing unit (CPU) and an NVIDIA GeForce RTX 5090 D graphics processing unit (GPU). The model is developed with Python 3.11, and the training process is implemented based on the PyTorch 2.10.0 framework with CUDA 12.8 support. Table 3 lists the hyperparameter settings for network training. During the training procedure, the adaptive moment estimation (Adam) optimizer and the ReduceLROnPlateau learning rate scheduler are adopted. The learning rate is shrunk to 0.5 times its original value once the validation set loss fails to drop for ten consecutive epochs. Accordingly, stable model convergence can be promoted.

To mitigate overfitting, an early-stopping strategy was employed. The validation loss was tracked throughout training, and training was terminated if the validation loss did not decrease for 15 consecutive epochs, with the model weights restored to those of the best-performing epoch. The maximum number of epochs was set to 100 as an upper bound.

For the multi-class event recognition task, four evaluation metrics, including accuracy, precision, recall, and F1-score, are adopted to assess the performance of the proposed model. Each single category is defined as follows.

(18) $$\mathrm{Accuracy}=\frac{\mathrm{TP}+\mathrm{TN}}{\mathrm{TP}+\mathrm{TN}+\mathrm{FP}+\mathrm{FN}}$$

(19) $$\mathrm{Precision}=\frac{\mathrm{TP}}{\mathrm{TP}+\mathrm{FP}}$$

(20) $$\mathrm{Recall}=\frac{\mathrm{TP}}{\mathrm{TP}+\mathrm{FN}}$$

(21) $$F1=2\times \frac{\mathrm{Precision}\times \mathrm{Recall}}{\mathrm{Precision}+\mathrm{Recall}}$$

where TP denotes the number of samples of a certain class that are correctly predicted as this class; FP denotes the number of samples from other classes that are incorrectly predicted as this class; TN denotes the number of samples not belonging to this class that are correctly predicted as non-this class; FN denotes the number of samples of this class that are incorrectly predicted as other classes. Accuracy represents the proportion of correctly classified samples among all classes. Precision represents the proportion of samples predicted as a given class by the model that belong to this class. Higher precision indicates fewer false positives. Recall denotes the proportion of actual samples of a class that are identified correctly. Higher recall means fewer false negatives. The F1-score is the harmonic mean of precision and recall, which can comprehensively reflect the balanced performance of the model in suppressing false positives and reducing false negatives. For the overall multi-class results, this study adopts Macro-averaging for the precision, recall and F1-score of each category, denoted as Macro-Precision, Macro-Recall and Macro-F1, respectively.

In addition to the metrics defined above, the number of model parameters and the single-batch inference time are adopted as efficiency metrics. The inference time was measured as follows. Each timing session began with 100 warm-up runs, and the reported latency was taken as the median of 500 independently timed runs. All latency values reflect the pure forward-propagation cost of the network. For MTFNet, the measured latency therefore covers the FFT operations of the frequency-domain branch, which are integral to the network. For GPU measurements, torch.cuda.synchronize() was called before and after the timed region, and CPU timings were obtained with a fixed number of threads. Unless otherwise specified, the inference time reported here refers to the single-batch latency at a batch size of 128 measured on the RTX 5090 D workstation.

### 4.2. Comparative Experiments Against Alternative Algorithms

To systematically evaluate the performance of MTFNet in the multi-type event recognition task of DAS, representative traditional machine learning methods and classical deep learning models are selected as baselines for comparison in this study, including SVM, 1D-CNN [8], temporal convolutional network (TCN) [26], MS 1D-CNN [15], convolutional bidirectional long short-term memory network (CNN-BiLSTM) [27], as well as the multimodal fusion-based Φ-OTDR signal recognition method proposed by Zhang et al. [20] (denoted as Zhang’s method hereafter). To ensure fair comparison, all models are trained and tested under identical data partitioning schemes, training strategies and hardware environments. In addition to the classification metrics of Accuracy, Macro-Precision, Macro-Recall and Macro-F1, the number of model parameters and the single-batch inference time are adopted as efficiency metrics. The timing protocol is detailed in Section 4.1. The performance of each method is comprehensively evaluated from three dimensions: recognition accuracy, model complexity and inference efficiency. The overall recognition results of all methods on the test set are presented in Table 4. It can be observed that MTFNet achieves the optimal performance across all overall evaluation metrics. Its accuracy, Macro-Precision, Macro-Recall and Macro-F1 reach 99.55%, 99.52%, 99.56% and 99.54%, respectively. This demonstrates that the proposed multi-scale time–frequency modeling strategy can effectively extract discriminative information from DAS vibration signals. The accuracy and Macro-F1 of SVM are 82.30% and 82.08%, which are considerably lower than those of deep learning methods. This is mainly because SVM relies on manually designed features and cannot adequately characterize the nonlinear differences among complex vibration events. By adopting an end-to-end convolutional architecture to automatically learn local temporal features, 1D-CNN improves the accuracy to 91.46%. TCN expands the temporal receptive field via dilated convolution. It increases the accuracy to 93.92%. MS 1D-CNN captures disturbance information at different time scales through parallel multi-scale convolutions. It achieves an accuracy of 95.36%. CNN-BiLSTM further introduces bidirectional temporal context modeling, lifting the accuracy to 95.98%. Compared with the above methods mainly relying on time-domain modeling, Zhang’s method achieves superior recognition performance by introducing STFT time–frequency diagrams and the Transformer attention fusion mechanism, with accuracy and Macro-F1 of 98.78% and 98.73%, respectively. This reveals that frequency-domain information plays a vital role in DAS event recognition. Nevertheless, this method requires constructing two-dimensional STFT time–frequency diagrams and simultaneously incorporates 1D-CNN, 2D-CNN and Transformer encoder, leading to relatively high model complexity and computational cost. MTFNet directly operates on the one-dimensional phase-difference signal and does not build a 2D time–frequency diagram. The time-domain branch obtains the local disturbance pattern, and in the frequency-domain branch, complementary frequency features are extracted by multi-scale spectrum analysis; a lightweight fusion module is employed to achieve time–frequency interaction. MTFNet has increased the accuracy by 0.77 percentage points and the Macro-F1 by 0.81 percentage points over Zhang’s method. At the same time, the single-batch inference time has been reduced from 3.46 ms to 2.30 ms, and the number of parameters is only 0.12 M. MTFNet has good trade-offs among recognition accuracy, model compactness, and inference speed, according to the above experiments. Notably, the SVM baseline runs on the CPU, and its latency includes handcrafted feature extraction. Its 21.60 ms is therefore not directly comparable with the GPU-based timings of the neural models. In contrast, the latency comparison among the deep learning models was conducted on the same GPU and is thus fair.

To further explore the recognition performance of all the models in different categories of events, Table 5 shows their F1-scores for the five types of events. It can be seen that Facility Noise originates from the continuous steady-state operation of the mobile power station, and its vibration is coupled to the optical fiber cable through the ground and wall, exhibiting stable periodic excitation and distinct narrowband spectral features; therefore, all models achieved a high F1-score for this class. Zhang’s method and MTFNet both reach 100.00%. The Wind, Sawing and Walking events are more likely to have background noise, fluctuating vibration intensity and signal non-stationarity; thus, they are more difficult to recognize. For example, the F1-scores of 1D-CNN for Wind and Walking are only 88.38% and 87.60%, respectively. Thus, single-scale temporal convolution has a relatively weak ability to distinguish complex disturbance patterns. Although TCN strengthens temporal modeling capacity, its F1-score on the Sawing class is merely 88.32%, which demonstrates that modeling relying solely on the time dimension cannot sufficiently characterize complex broadband spectral variations. CNN-BiLSTM has an F1-score of 99.02% for the Hammering class. Notably, the performance of CNN-BiLSTM on Sawing and Walking can be improved further. By introducing time–frequency diagrams and an attention fusion mechanism, Zhang’s method achieves evident improvements on difficult categories. Its F1-scores for Wind, Facility Noise, Hammering, Sawing and Walking are 98.60%, 100.00%, 99.12%, 96.92% and 99.00%, respectively. This further proves that time–frequency information fusion is of great significance for DAS vibration event recognition.

MTFNet achieves the best or tied-best performance across all categories. Its F1-scores reach 99.26%, 100.00%, 99.70%, 98.93%, and 99.80% for Wind, Facility Noise, Hammering, Sawing, and Walking events, respectively.

Particularly for the challenging Sawing class, MTFNet outperforms CNN-BiLSTM by 7.67 percentage points and surpasses Zhang’s method by 2.01 percentage points. This reveals that the multi-scale frequency-domain branch can effectively capture transient frequency variations and steady spectral structures within complex vibration events, and therefore enhance the discriminative capacity among different classes.

**Figure 5:**
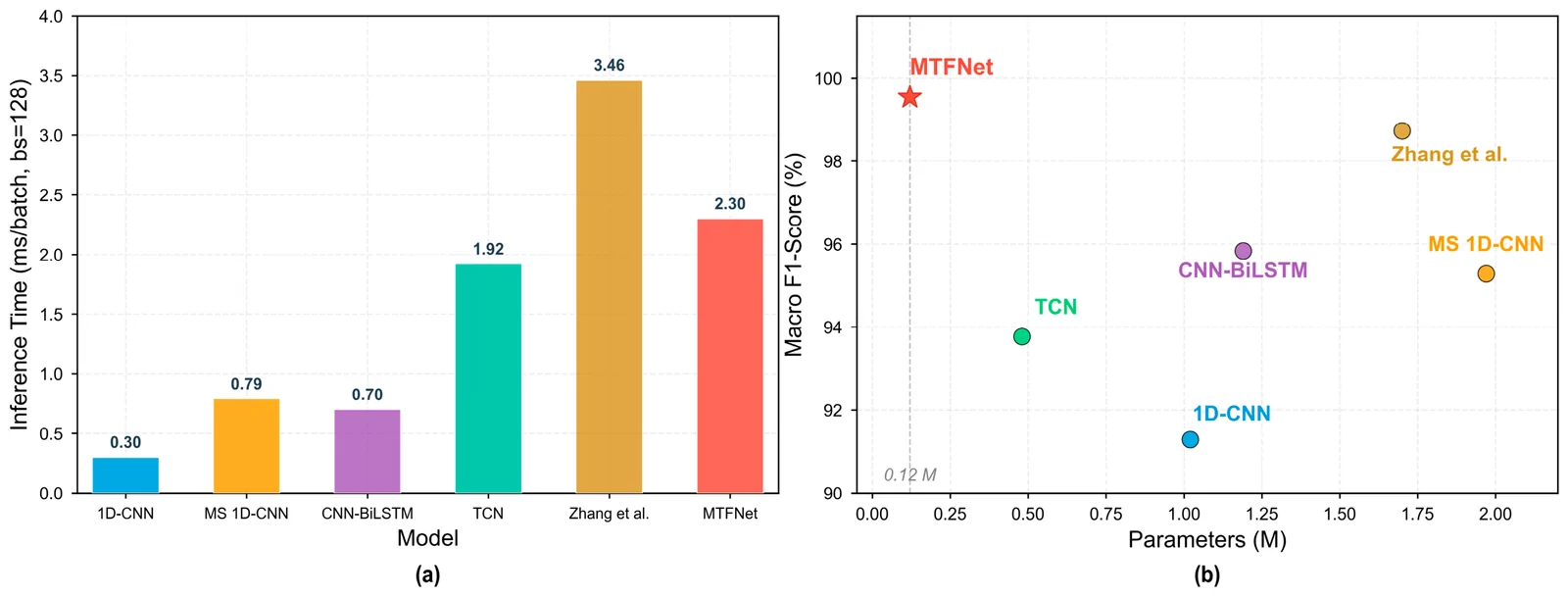
Computational efficiency analysis. (**a**) Comparison of single-batch inference time for deep learning models (1D-CNN [8], TCN [26], MS 1D-CNN [15], CNN-BiLSTM [27], Zhang’s method [20], and MTFNet); (**b**) relationship between parameter count and Macro-F1.

Other than recognition accuracy, model complexity and inference speed also need to be considered in the engineering application. Figure 5 shows the computational efficiency of the various deep learning models. Among them, Figure 5a shows the single-batch inference time of each model, and Figure 5b shows how model parameter count relates to Macro-F1. It can be seen that 1D-CNN has the shortest inference time at 0.30 ms, but its Macro-F1 is only 91.29%, showing relatively weak recognition performance. The inference time of CNN-BiLSTM is 0.70 ms and that of MS 1D-CNN is 0.79 ms; both models have a Macro-F1 of less than 96%. TCN has 0.48 M parameters and an inference time of 1.92 ms. In each stacked residual block, TCN performs convolutions over the full-length sequence without downsampling. Therefore, its overall computational cost remains higher than that of plain convolutional models with progressive pooling, despite its small parameter count. Zhang’s method achieves a Macro-F1 of 98.73% and an inference time of 3.46 ms. This method extracts multimodal features using parallel one- and two-dimensional CNN branches, then fuses them through a three-layer LSTM and a three-layer eight-head Transformer encoder. The recurrent and self-attention computations account for the major inference cost. MTFNet has a higher Macro-F1 of 99.54% and only 0.12 M parameters; it also has a single-batch inference speed of 2.30 ms. The batch-1 latencies in Table 4 follow the same ranking, with MTFNet at 1.84 ms per sample, still faster than Zhang’s method (2.25 ms). It should be noted that the 2.30 ms single-batch inference time measures only the network forward-propagation cost. In practical continuous monitoring, each decision is made on a non-overlapping 2 s frame of the signal stream. The total detection latency of a single event is dominated by the frame duration and is on the order of seconds, which is consistent with the reporting-cycle requirements of perimeter monitoring. MTFNet has a good overall trade-off among recognition accuracy, parameter count and inference speed, and its lightweight design shows potential for deployment on resource-constrained hardware.

To assess the deployability of MTFNet on resource-constrained hardware, its inference latency was additionally measured on a notebook computer equipped with an NVIDIA GeForce RTX 3070 Laptop GPU. The latency was also measured on the notebook’s CPU (Intel Core i7-11800H). Both measurements followed the timing protocol described in Section 4.1. The results are summarized in Table 6.

As shown in Table 6, MTFNet maintains a batch-1 inference latency of 3.76 ms on the notebook GPU and 4.61 ms even on the notebook CPU, both far below the 2 s frame duration, which therefore meets the real-time requirements of perimeter monitoring without dedicated GPU hardware.

**Figure 6:**
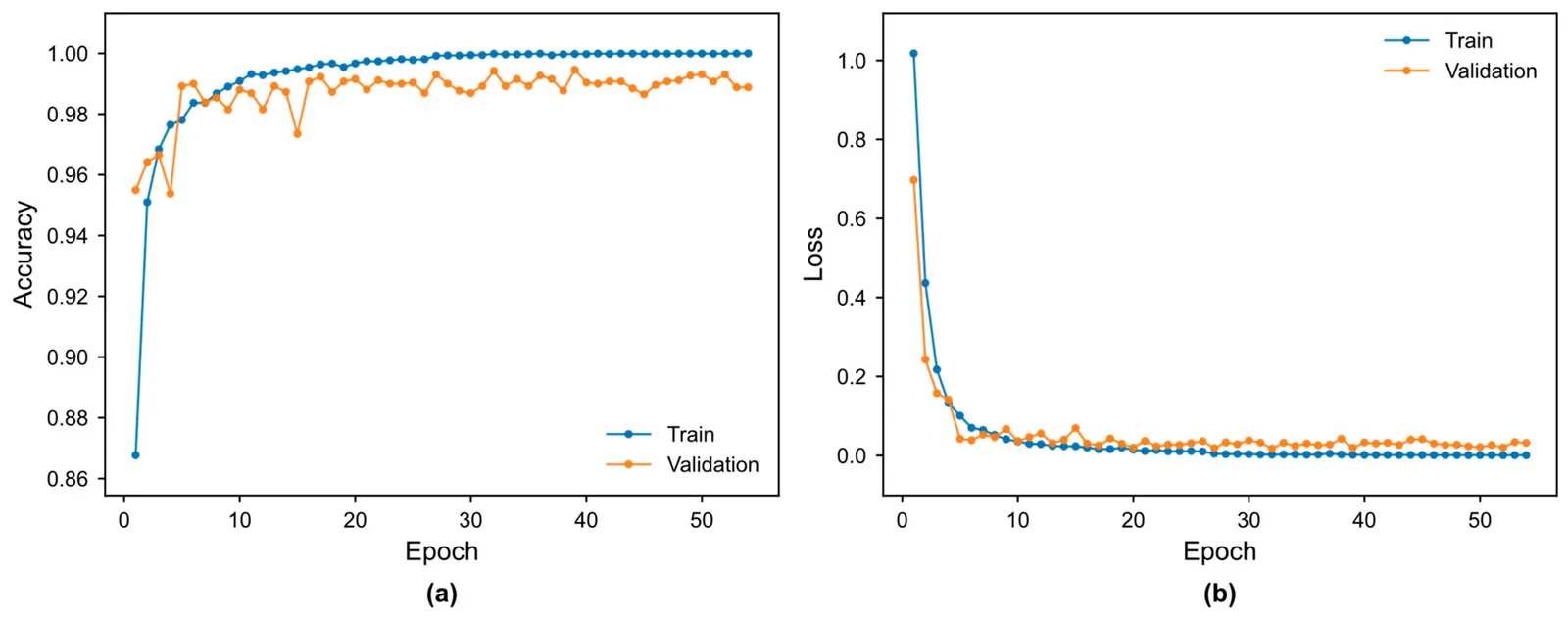
Accuracy and loss curves of MTFNet for training and validation datasets. (**a**) Accuracy curves; (**b**) loss curves.

To verify the training stability and convergence characteristics of MTFNet, Figure 6 plots the curves of Accuracy and Loss varying with epoch for the model on the training set and validation set. It can be observed that the model accuracy rises rapidly and the loss drops sharply in the early training stage, and the accuracy exceeds 99% at approximately the 25th epoch. Afterwards, the curves of the training set and validation set maintain high consistency all the time; the loss curves decline steadily and converge gradually without obvious oscillation or divergence. After training completes, the accuracy on the validation set remains steadily at a high level. Meanwhile, the performance gap between the training set and validation set is small. These results demonstrate that MTFNet possesses favorable convergence performance and generalization ability, and no severe overfitting occurs. The horizontal axis of Figure 6 represents the actual training process. Training terminates at epoch 54 due to the early-stopping strategy described in Section 4.1, rather than the preset maximum of 100 epochs. The best validation performance was obtained at epoch 39.

**Figure 7:**
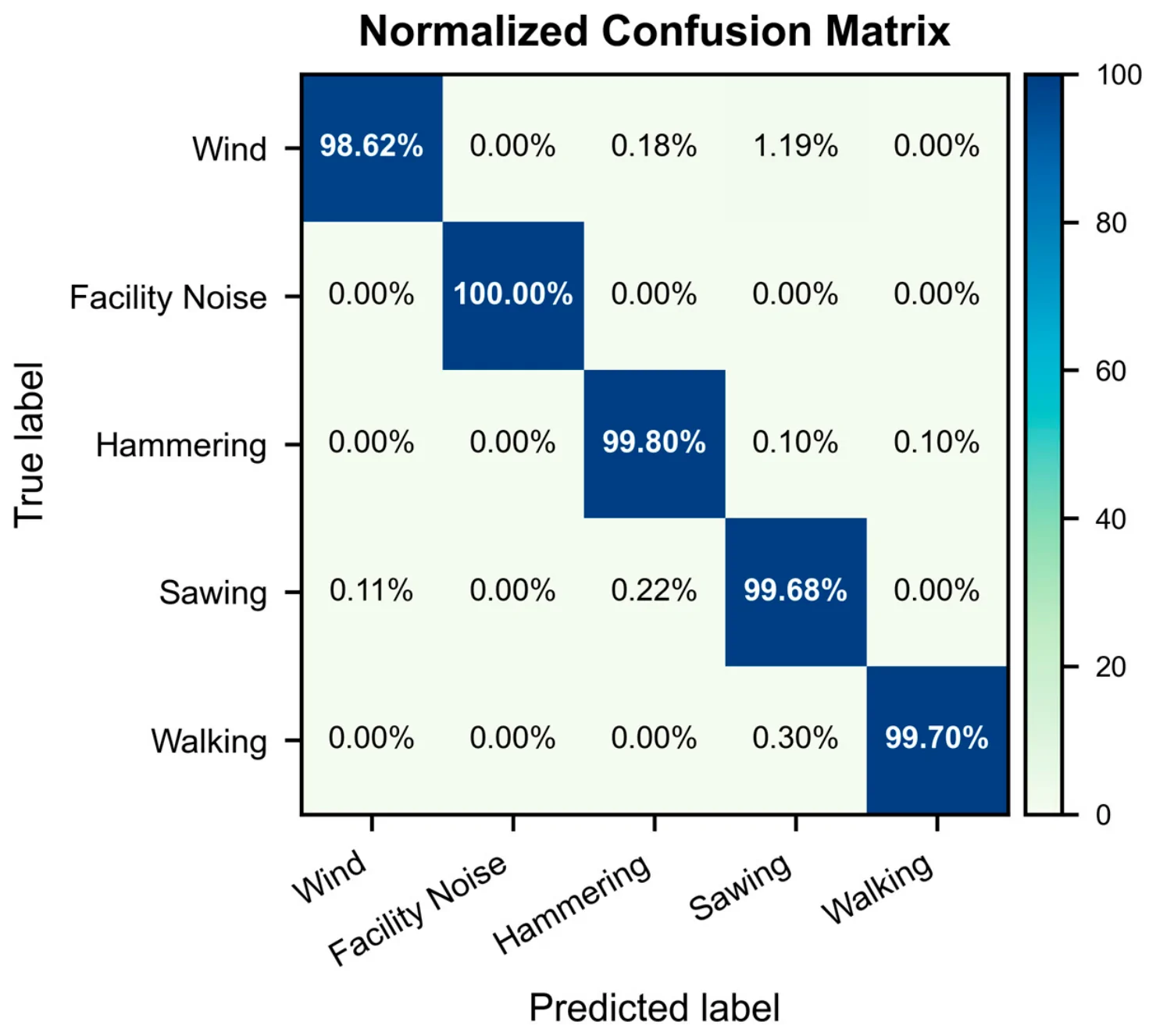
Normalized confusion matrix of MTFNet on the test set.

Figure 7 presents the normalized confusion matrix yielded by MTFNet on the test dataset. The recognition accuracy for all five vibration event categories exceeds 98.60%. Facility Noise detection achieves 100.00%, while Hammering, Sawing and Walking reach 99.80%, 99.68% and 99.70%, respectively. A small number of misclassified samples mainly occur between Wind interference and Sawing events. This phenomenon can be attributed to the overlapping energy distribution in the low-frequency band of the two types of signals. Overall, low inter-class confusion ratios are maintained across all categories. The results further verify the stability and robustness of MTFNet for multi-class vibration event recognition tasks.

In summary, compared with traditional machine learning schemes and mainstream deep learning approaches, MTFNet exhibits prominent advantages in recognition accuracy, category balance, model complexity and inference efficiency.

### 4.3. Ablation Experiments

To verify the contribution of each component in the proposed MTFNet to the model’s recognition performance, five model variants are designed for ablation experiments in this study. Time-Only retains only the time-domain feature-extraction branch; Freq-Only retains only the frequency-domain feature-extraction branch; Time-Freq (Single-FFT) integrates the time-domain branch and a single-scale FFT frequency-domain branch with a fixed frequency-domain window length of 256; Time-Freq (Direct-Concat) retains both branches and the multi-scale spectral analysis, but removes the fusion block. The two 96-dimensional features are directly concatenated and fed into the classification head. MTFNet denotes the complete model proposed in this study. The overall classification performance of different variants is listed in Table 7, and the category-wise classification results are presented in Table 8.

As shown in Table 7, the overall performance of the five models exhibits a gradual improvement trend, indicating that time-domain modeling, frequency-domain modeling, time–frequency fusion and multi-scale spectral analysis all make positive contributions to the final recognition results. The accuracy of the Time-Only model reaches 96.53%, demonstrating that the time-domain branch can effectively extract temporal disturbance features of events. Nevertheless, its precision for the Sawing category is merely 86.73%, and the recall values of Wind and Walking are only 92.00% and 92.51%, respectively. This reveals that relying solely on time-domain information limits the model’s capability to distinguish events with similar temporal patterns.

The Freq-Only setup exhibits an accuracy of 97.61%, accounting for a 1.08-percentage-point increment over Time-Only. This demonstrates that frequency-domain features bring superior class separability. Specifically, the recall of Wind-disturbance samples rises from 92.00% to 98.44%, and the precision of Sawing-event detection increases from 86.73% to 91.37%. It can be concluded that spectral features reduce misjudgments targeting the easily confused Sawing category. Even so, the recall of Walking events drops to 91.22%, inferior to the Time-Only baseline. The above results reveal that frequency-domain modeling alone cannot fully depict events with obvious time-evolving characteristics.

Furthermore, the Single-FFT model outperforms both single-branch networks by merging time-domain and single-scale frequency-domain features, which proves the complementarity between temporal and spectral information. For Hammering events under the above configuration, precision, recall and F1-score are respectively obtained as 99.70%, 100.00%, and 99.85%, and the recall of Walking-related vibration rises to 93.71%. Nevertheless, the precision for Sawing events remains merely 91.04%. As indicated by the above-mentioned results, single-scale frequency-domain analysis fails to fully characterize subtle spectral discrepancies within complicated vibration signals.

To isolate the contribution of the fusion block, the Direct-Concat variant was constructed. In this variant, the 96-dimensional features of the two branches are concatenated and fed directly into the classification head. As shown in Table 7, removing the fusion block decreases the accuracy from 99.55% to 99.22%. This indicates that the lightweight fusion block contributes 0.33 percentage points to the final accuracy at a negligible parameter cost. Meanwhile, the Direct-Concat variant still outperforms the Single-FFT variant by 1.06 percentage points. This confirms that the gain from multi-scale spectral analysis holds regardless of the fusion strategy.

The complete MTFNet yields the optimal metrics among all ablation variants. Its accuracy and Macro-F1 hit 99.55% and 99.54%, growing by 1.39 and 1.46 percentage points compared with Single-FFT. Such results validate the effectiveness of the multi-scale spectral-analysis strategy. From the category-wise perspective, MTFNet delivers more balanced recognition performance across the five vibration classes. For Facility Noise, the precision, recall and F1-score all reach 100.00%. This is mainly attributed to the fact that Facility Noise is generated by a continuously operating mobile power station, resulting in relatively stable vibration patterns and prominent periodic components in the spectrum, which provide high class separability. The precision and F1-score of Sawing events increase to 98.19% and 98.93%, while Walking-event recall and F1-score reach 99.70% and 99.80%. As revealed by the aforementioned findings, collaborative multi-scale time–frequency modeling extracts complementary discriminative features at multiple resolutions, and accordingly strengthens the model’s capability to distinguish highly similar vibration incidents.

**Figure 8:**
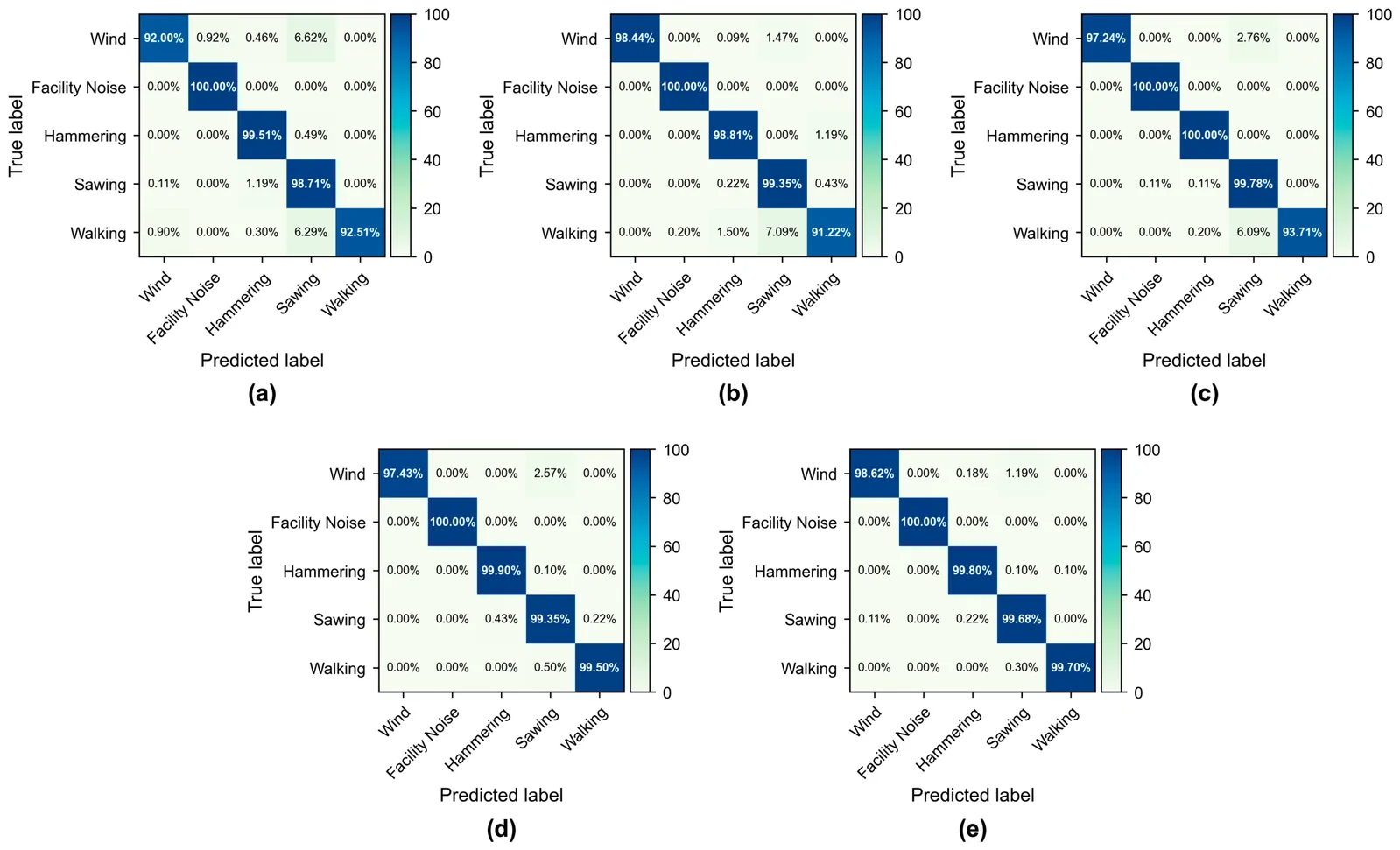
Confusion matrices of different ablation models. (**a**) Time-Only; (**b**) Freq-Only; (**c**) Time-Freq (Single-FFT); (**d**) Time-Freq (Direct-Concat); (**e**) MTFNet.

Figure 8 illustrates the confusion matrices yielded by each ablation variant, with the aim of exploring how different network configurations affect easily confused categories. For the Time-Only model, 6.62% of Wind-disturbance samples and 6.29% of Walking samples are misclassified as Sawing events, thereby primarily accounting for the unsatisfactory precision of the Sawing class. The introduction of frequency-domain features alleviates inter-category confusion to some extent, yet the Single-FFT model still yields a Sawing-class precision of merely 91.04%. For the Direct-Concat variant, 2.57% of Wind samples and 0.50% of Walking samples are still misclassified as Sawing. The resulting precision for the Sawing class is 96.44%. By comparison, MTFNet greatly cuts down misclassification ratios. The proportion of Walking samples wrongly recognized as Sawing drops to 0.30%, which further verifies that multi-scale frequency-domain modeling and time–frequency fusion can be conducive to mitigating category confusion.

**Figure 9:**
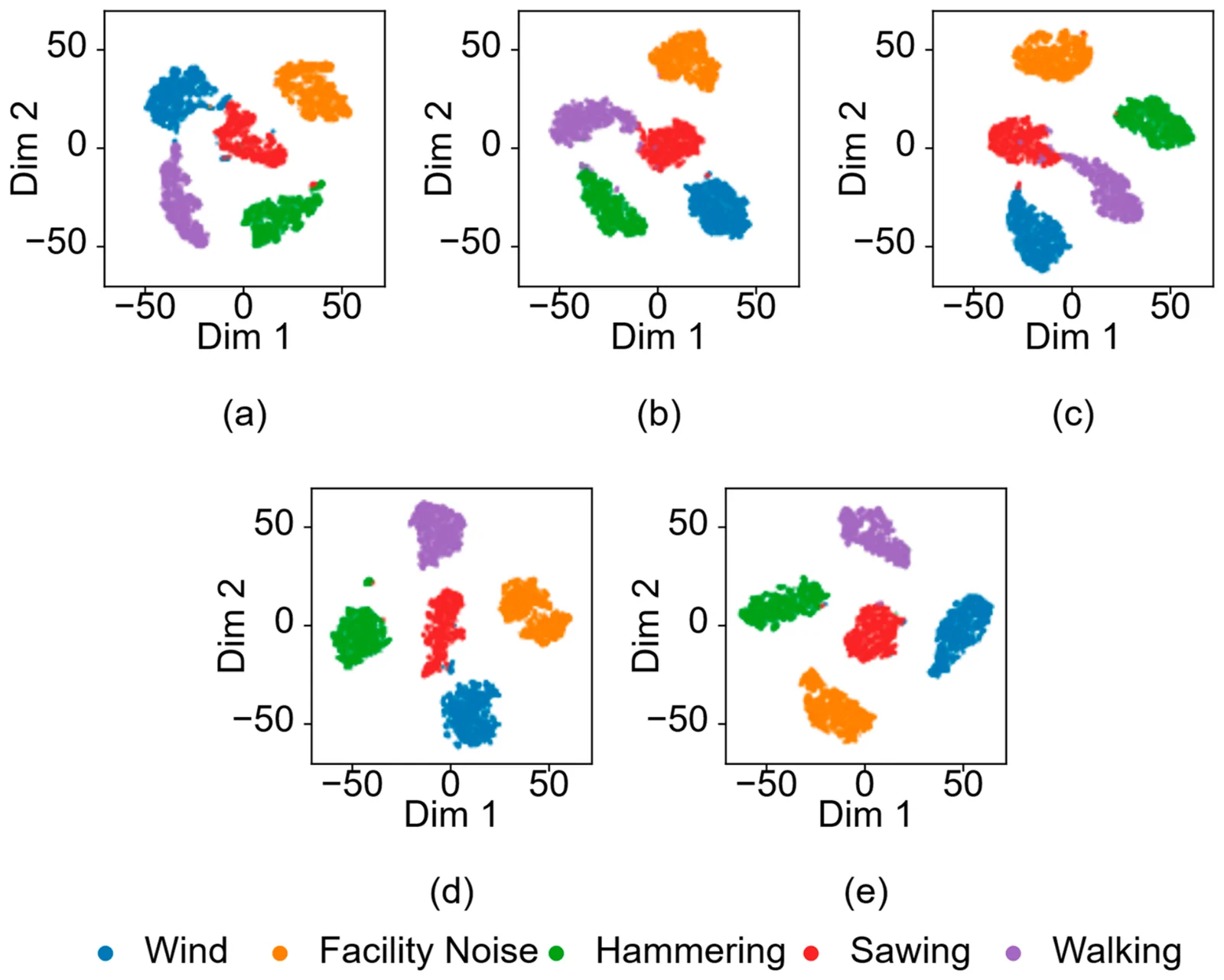
t-SNE visualization of the last-layer features of different ablation models. (**a**) Time-Only; (**b**) Freq-Only; (**c**) Time-Freq (Single-FFT); (**d**) Time-Freq (Direct-Concat); (**e**) MTFNet.

Figure 9 depicts t-distributed stochastic neighbor embedding (t-SNE) visualization results of the last-layer features extracted from different ablation models. It can be observed that Time-Only is able to form basic clusters; however, significant overlaps remain among Wind, Walking, and Sawing, indicating that relying solely on time-domain features limits the model’s capability to distinguish complex non-stationary disturbance events. Freq-Only achieves improved inter-class separation. Specifically, the Facility Noise class exhibits a higher degree of aggregation in the feature space because it is mainly caused by the continuous operation of the mobile power station and thus possesses relatively stable periodic spectral characteristics. Nevertheless, the Walking class remains relatively scattered, suggesting that single frequency-domain modeling is insufficient to fully capture the temporal variation patterns of walking events. Time-Freq (Single-FFT) further improves the clarity of class boundaries by integrating time-domain and frequency-domain information, but overlaps still exist between some similar categories, demonstrating that single-scale spectral analysis lacks sufficient multi-resolution representation capability for complex disturbance events. For the Time-Freq (Direct-Concat) variant, the five event categories still form generally well-separated clusters, which is consistent with its accuracy of 99.22%. Nevertheless, its intra-class compactness and inter-class boundaries degrade to some extent compared with the complete model. The Walking samples remain relatively scattered. A small subset of Hammering samples forms a satellite cluster detached from its main cluster. A few Wind samples drift toward the Sawing cluster, which matches the confusion-matrix results, in which the misclassifications of this variant are concentrated between Wind and Sawing. This indicates that directly concatenating the two-branch features merely juxtaposes the time-domain and multi-scale frequency-domain representations. By contrast, the lightweight fusion block further calibrates the interaction between the two domains and sharpens the class boundaries. In contrast, the features learned by MTFNet exhibit more compact intra-class distributions and clearer inter-class separations in the two-dimensional space, particularly showing more distinct boundaries between easily confused categories such as Sawing and Walking. Meanwhile, MTFNet further enhances the separability between periodic events, such as Facility Noise, and non-stationary events, such as Sawing and Walking. These results demonstrate that the proposed multi-scale time–frequency fusion architecture can learn more discriminative feature representations through the collaborative modeling of temporal and multi-scale frequency-domain features, thereby effectively improving the discrimination capability among different perimeter security events.

In brief, the following conclusions can be drawn from the ablation experiments. The time-domain branch captures time-evolving vibration patterns; the frequency-domain branch enhances separability on the basis of frequency components; time–frequency fusion improves the overall feature-representation capacity; multi-scale frequency-domain modeling acquires complementary information under multiple resolutions and reduces confusion between analogous categories. The indispensability and coordinated effectiveness of each functional module within MTFNet can be confirmed by the gradual performance gap in five model variants.

### 4.4. Analysis of Noise Robustness Under Different Signal-to-Noise Ratios

During the practical deployment of DAS systems, optical fibers deployed along the monitoring line are inevitably affected by environmental disturbances, such as wind, rain, traffic-induced vibrations, and circuit noise from acquisition devices. These factors significantly reduce the signal-to-noise ratio (SNR) of acquired signals and degrade the recognition accuracy of intrusion events. Therefore, evaluating the model’s recognition performance under varying noise intensities is crucial for assessing its robustness against additive noise. In this study, the moving average method was adopted to estimate the signal power and noise power, and the SNR is calculated as:

(22) $$\mathrm{SNR}=10\cdot \log_{10}\left(\frac{P_{s}}{P_{n}+\varepsilon }\right)$$

where *P_s_* and *P_n_* denote the estimated average signal power and average noise power, respectively, and *ε* is a small positive constant to avoid the denominator approaching zero. Statistics show that the average SNR of the original dataset is 11.48 dB.

**Figure 10:**
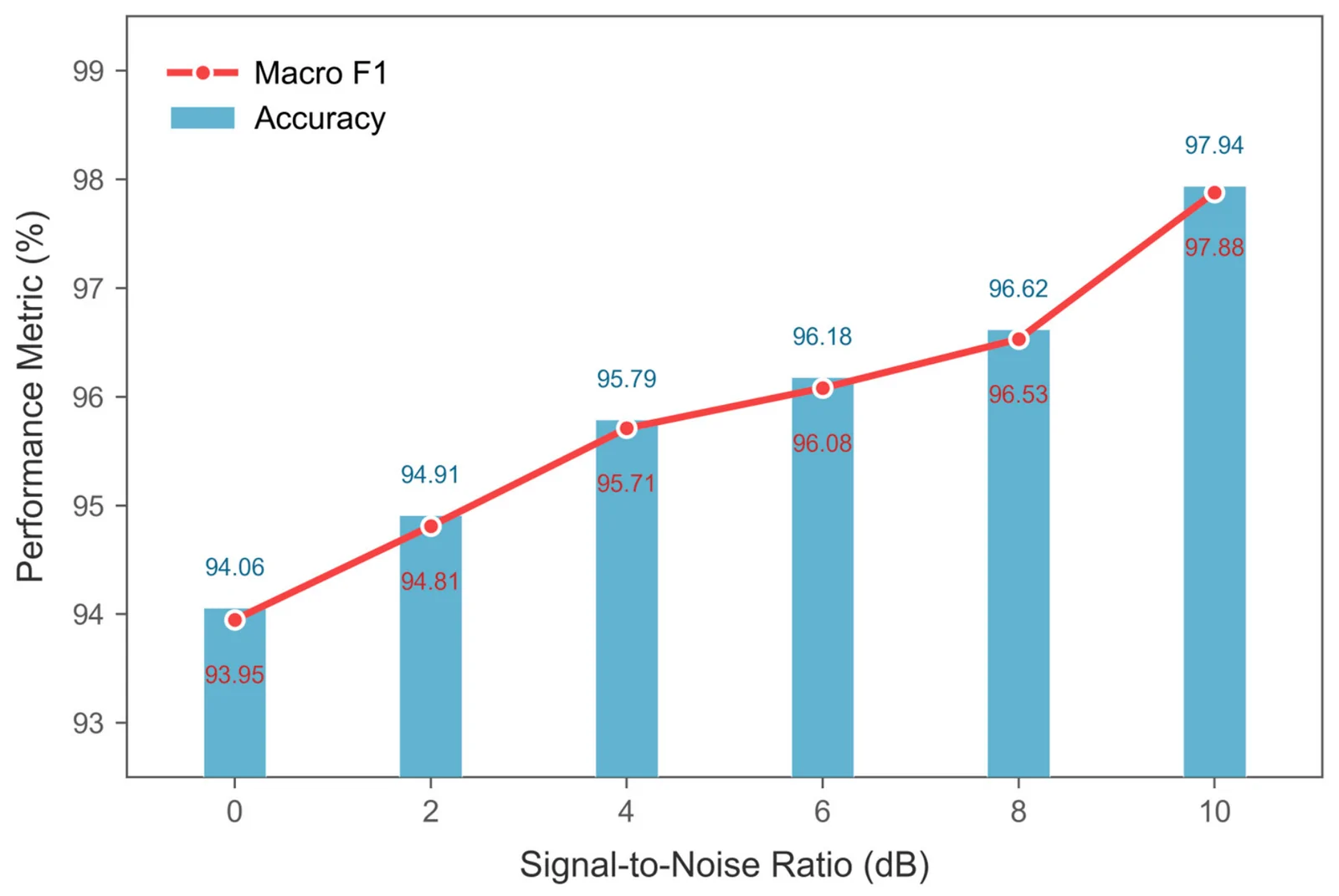
Accuracy and Macro-F1 as functions of SNR.

**Figure 11:**
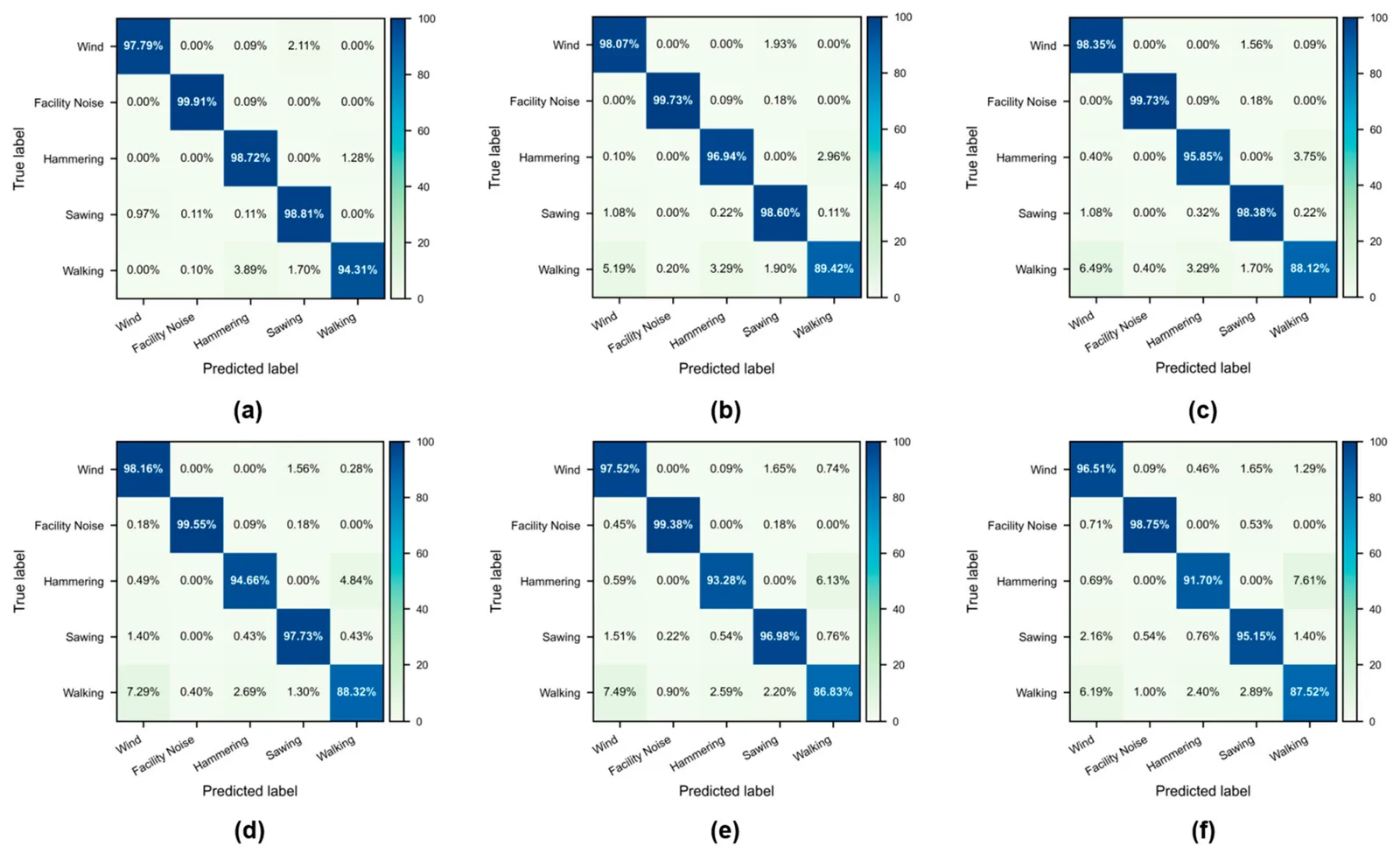
Normalized confusion matrices under different SNR conditions. (**a**) 10 dB; (**b**) 8 dB; (**c**) 6 dB; (**d**) 4 dB; (**e**) 2 dB; (**f**) 0 dB.

To further investigate the recognition capability of MTFNet under complex noise environments and lower SNR conditions, additive white Gaussian noise (AWGN) was added to the original test set signals to construct six groups of test data with SNR levels of 10, 8, 6, 4, 2, and 0 dB. Inference was performed using the same trained model weights to evaluate the robustness of the proposed method against additional noise interference. The overall recognition results are presented in Table 9. The variation trends of accuracy and Macro-F1 with different SNR levels are illustrated in Figure 10, and the normalized confusion matrices under each SNR condition are shown in Figure 11. It should be noted that Facility Noise in this study refers to a type of background vibration event generated by the operation of the mobile power station, whereas the noise interference mentioned in this section refers to the externally added AWGN used specifically for robustness evaluation. Therefore, these two terms represent different concepts.

As can be observed from Table 9, as the SNR gradually decreases from 10 dB to 0 dB, the accuracy and Macro-F1 of MTFNet show a steady declining trend, while the overall performance remains at a high level. At an SNR of 10 dB, the model has an accuracy of 97.94% and a Macro-F1 of 97.88%. The reductions relative to the original dataset are only 1.61 and 1.66 percentage points, respectively. At an SNR of 6 dB, both metrics still remain at 96.18% and 96.08%, and the performance drop is less than 3.5 percentage points. Even at a high noise level of 0 dB, the accuracy and Macro-F1 remain at 94.06% and 93.95%, and the drop is less than 5.6 percentage points. Thus, MTFNet maintains stable recognition performance under AWGN interference. This robustness stems from the proposed multi-window-length parallel FFT frequency-domain branch.

Short windows capture the spectra of noise-masked transient impulses, while long windows extract steady narrowband frequency components that are less disturbed by noise. The complementarity of multi-scale spectra reduces the susceptibility to noise compared with single-scale analysis. Such a robustness mechanism against AWGN is difficult to achieve for models that rely solely on single-domain or single-scale spectral features.

Further analysis of the confusion matrices for each category reveals that Facility Noise maintains an extremely high recognition rate under all noise conditions. This robustness can be attributed to the periodic excitations generated by the steady-state operation of the mobile power station, as well as their distinct narrowband spectral characteristics. Even under low signal-to-noise ratio conditions, these discriminative frequency components can still be effectively captured by the multi-scale frequency-domain branch. Sawing and Wind are also quite stable, and their recognition rates are still at 95.15% and 96.51% respectively at 0 dB. Therefore, it can be seen that the multi-scale time–frequency fusion architecture can extract complementary features at different frequency resolutions and thus reduce the impact of noise in local frequency bands. Hammering and Walking are relatively more sensitive to noise. As the SNR decreases from 10 dB to 0 dB, the recognition rate of Hammering drops from 98.72% to 91.70%, and the misclassified samples are mainly recognized as Walking. At the same time, the recognition rate of Walking drops to 87.52%, and this category is more likely to be confused with Wind and Hammering. Hammering and Walking are both low-frequency transient vibration phenomena, and as a result, their time-domain waveforms and spectral patterns are similar. Performance degradation remains moderate for most categories. However, Hammering and Walking are more sensitive to the added noise, as analyzed above. Even at 0 dB, the diagonal recognition rates of all five types of events are still above 87.50%. Overall, MTFNet maintains stable recognition performance across all tested noise levels. Its robustness can be attributed to the multi-branch time–frequency collaborative architecture, in which the time-domain branch captures local disturbance patterns and helps suppress local noise fluctuations, while the frequency-domain branch extracts complementary discriminative features across multiple frequency scales. This design reduces the model’s reliance on single-domain or single-scale information and improves robustness against AWGN-type interference.

As shown in Table 10, the accuracy of Time-Only drops by 8.69 percentage points, from 94.72% at 10 dB to 86.03% at 0 dB. In contrast, Freq-Only declines by 5.26 percentage points, from 95.56% to 90.30%, indicating that frequency-domain features are more robust to AWGN than time-domain features. Replacing single-scale spectral analysis with multi-scale spectral analysis further narrows the decline, from 4.72 percentage points for the Single-FFT variant to 4.25 percentage points for the Direct-Concat variant. This confirms that the robustness gain from multi-scale spectral analysis holds regardless of the fusion strategy. Among all variants, the complete MTFNet exhibits the smallest decline of 3.88 percentage points, and its accuracy at 0 dB remains 94.06%. This value is 1.57 percentage points above that of Direct-Concat and 8.03 percentage points above that of Time-Only. Notably, the advantage of MTFNet over Time-Only grows from 3.22 percentage points at 10 dB to 8.03 percentage points at 0 dB. This finding suggests that the collaborative modeling of temporal and multi-scale spectral features is particularly beneficial under strong noise, since noise corruption in local frequency bands can be offset by the remaining scales and by the time-domain branch.

## 5. Conclusions

This study proposes the multi-scale time–frequency network MTFNet for DAS-based perimeter security event recognition. Taking normalized one-dimensional phase-difference sequences as input, the presented framework extracts discriminative features cooperatively via the time-domain branch and multi-scale frequency-domain branch, and conducts joint modeling of temporal-spectral features with a lightweight fusion module. Unlike algorithms that require two-dimensional time–frequency spectrogram generation, MTFNet avoids the explicit spectrogram construction step. It reduces computational overhead and enhances the representation capability for complex vibration incidents.

Experimental results on the self-collected five-class DAS event dataset demonstrate that MTFNet achieves an accuracy, Macro-Precision, Macro-Recall, and Macro-F1 of 99.55%, 99.52%, 99.56%, and 99.54%, respectively, outperforming SVM, 1D-CNN, TCN, MS 1D-CNN, CNN-BiLSTM, and the multimodal fusion method proposed by Zhang et al. [20]. Compared with Zhang’s method, MTFNet improves the accuracy and Macro-F1 by 0.77 and 0.81 percentage points, respectively. Its single-batch inference latency decreases from 3.46 ms to 2.30 ms, with the total number of model parameters as low as 0.12 M. These results demonstrate that MTFNet achieves favorable trade-offs among classification precision, model compactness, and inference efficiency.

Ablation experiments further verify the contributions of time-domain modeling, frequency-domain modeling and multi-scale spectrum analysis to the performance improvement of the model. Noise robustness experiments demonstrate that MTFNet maintains an accuracy of 94.06% when the SNR drops to 0 dB. This indicates robustness against additive white Gaussian noise at low SNR levels. Robustness under DAS-specific degradation mechanisms and cross-scenario generalization remain to be verified.

Overall, MTFNet offers a lightweight and effective solution for real-time recognition of DAS perimeter security events. Future work will further investigate cross-scenario generalization, cross-device transfer, and robustness under complex low SNR conditions, so as to improve the applicability of the model in practical heterogeneous DAS deployment environments.

## Figures and Tables

**Table 1:** Distribution of dataset samples.

Category	Total	Training Set	Validation Set	Testing Set
Wind	5492	3834	570	1088
Facility Noise	5635	3941	571	1123
Hammering	5125	3587	526	1012
Sawing	4676	3292	457	927
Walking	5377	3901	474	1002

**Table 2:** Acquisition statistics of the dataset.

Category	Sessions	Positions	Operators	Samples
Wind	200	All wall-mounted sections	natural event	5492
Facility Noise	190	Wall-mounted sections	power station	5635
Hammering	240	15	3	5125
Sawing	235	8	3	4676
Walking	225	All buried sections	4	5377

**Table 3:** Hyperparameter configuration.

Hyperparameter	Value
Optimizer	Adam
Initial learning rate	0.0005
Learning rate adjustment method	ReduceLROnPlateau
Reduction factor	0.5
Patience	10
Batch size	128
Early-stopping patience	15
Maximum epochs	100
Loss function	Cross-entropy loss

**Table 4:** Overall comparison with other methods.

Model	Accuracy (%)	Macro- Precision (%)	Macro- Recall (%)	Macro-F1 (%)	Params (M)	Inference Time (ms), Batch 128	Inference Time (ms), Batch 1
SVM	82.30	83.15	81.81	82.08	-	21.60	0.25
1D-CNN	91.46	91.31	91.44	91.29	1.02	0.30	0.25
TCN	93.92	94.32	93.75	93.77	0.48	1.92	1.24
MS 1D-CNN	95.36	95.28	95.30	95.29	1.97	0.79	0.54
CNN-BiLSTM	95.98	96.09	95.93	95.84	1.19	0.70	0.65
Zhang et al. [20]	98.78	98.71	98.77	98.73	1.70	3.46	2.25
MTFNet	99.55	99.52	99.56	99.54	0.12	2.30	1.84

Note: The latency values at batch sizes of 128 and 1 were measured following the protocol in Section 4.1. The inference time of the SVM baseline was measured on the workstation CPU with a fixed number of threads, including the handcrafted feature-extraction step, whereas all deep learning models were measured on the RTX 5090 D GPU; the absolute latency values of the two groups are therefore not directly comparable.

**Table 5:** Comparison of F1-score for each event category.

Model	Wind (%)	Facility Noise (%)	Hammering (%)	Sawing (%)	Walking (%)
SVM	79.93	93.32	76.02	76.73	84.40
1D-CNN	88.38	99.24	91.55	89.67	87.60
TCN	93.18	99.82	95.79	88.32	91.73
MS 1D-CNN	92.46	99.64	93.87	94.05	96.43
CNN-BiLSTM	96.23	99.60	99.02	91.26	93.08
Zhang et al. [20]	98.60	100.00	99.12	96.92	99.00
MTFNet	99.26	100.00	99.70	98.93	99.80

**Table 6:** Inference latency of MTFNet on different hardware platforms.

Hardware Platform	Batch Size	Inference Latency (ms)
RTX 5090 D	128	2.30
RTX 5090 D	1	1.84
RTX 3070 Laptop (GPU, notebook)	128	6.97
RTX 3070 Laptop (GPU, notebook)	1	3.76
Notebook CPU (Intel Core i7-11800H)	1	4.61

**Table 7:** Overall performance comparison of ablation models.

Model	Accuracy (%)	Macro-Precision (%)	Macro-Recall (%)	Macro-F1 (%)
Time-Only	96.53	96.60	96.55	96.44
Freq-Only	97.61	97.54	97.56	97.49
Time-Freq (Single-FFT)	98.16	98.13	98.15	98.08
Time-Freq (Direct-Concat)	99.22	99.17	99.24	99.19
MTFNet	99.55	99.52	99.56	99.54

**Table 8:** Per-class performance of different ablation models.

Model	Event Type	Precision (%)	Recall (%)	F1-Score (%)
	Wind	99.01	92.00	95.38
	Facility Noise	99.12	100.00	99.56
Time-Only	Hammering	98.15	99.51	98.82
	Sawing	86.73	98.71	92.33
	Walking	100.00	92.51	96.11
	Wind	100.00	98.44	99.21
	Facility Noise	99.82	100.00	99.91
Freq-Only	Hammering	98.23	98.81	98.52
	Sawing	91.37	99.35	95.19
	Walking	98.28	91.22	94.62
	Wind	100.00	97.24	98.60
	Facility Noise	99.91	100.00	99.96
Time-Freq (Single-FFT)	Hammering	99.70	100.00	99.85
	Sawing	91.04	99.78	95.21
	Walking	100.00	93.71	96.75
	Wind	100.00	97.43	98.70
	Facility Noise	100.00	100.00	100.00
Time-Freq (Direct-Concat)	Hammering	99.61	99.90	99.75
	Sawing	96.44	99.35	97.87
	Walking	99.80	99.50	99.65
	Wind	99.91	98.62	99.26
	Facility Noise	100.00	100.00	100.00
MTFNet	Hammering	99.61	99.80	99.70
	Sawing	98.19	99.68	98.93
	Walking	99.90	99.70	99.80

**Table 9:** Recognition performance of MTFNet under different SNR conditions.

SNR (dB)	Accuracy (%)	Macro-F1 (%)	Decline in Accuracy (Percentage Points)	Decline in Macro-F1 (Percentage Points)
10	97.94	97.88	1.61	1.66
8	96.62	96.53	2.93	3.01
6	96.18	96.08	3.37	3.46
4	95.79	95.71	3.76	3.83
2	94.91	94.81	4.64	4.73
0	94.06	93.95	5.49	5.59

**Table 10:** Recognition accuracy of the ablation variants under different SNR conditions.

Model	10 dB	8 dB	6 dB	4 dB	2 dB	0 dB
Time-Only	94.72	94.20	93.07	91.75	89.91	86.03
Freq-Only	95.56	95.30	94.76	93.71	92.08	90.30
Time-Freq (Single-FFT)	96.06	95.79	95.44	94.60	93.25	91.34
Time-Freq (Direct-Concat)	96.74	96.33	95.71	95.05	93.34	92.49
MTFNet	97.94	96.62	96.18	95.79	94.91	94.06

## Data Availability

The data presented in this study are available on request from the corresponding author. The data are not publicly available due to the sensitive nature of the field experiments, which were conducted at a military-critical protection target site with restricted access and security protocols.
